# Complement-associated loss of CA2 inhibitory synapses in the demyelinated hippocampus impairs memory

**DOI:** 10.1007/s00401-021-02338-8

**Published:** 2021-06-25

**Authors:** Valeria Ramaglia, Mohit Dubey, M. Alfonso Malpede, Naomi Petersen, Sharon I. de Vries, Shanzeh M. Ahmed, Dennis S. W. Lee, Geert J. Schenk, Stefan M. Gold, Inge Huitinga, Jennifer L. Gommerman, Jeroen J. G. Geurts, Maarten H. P. Kole

**Affiliations:** 1 Department of Axonal Signaling, Netherlands Institute for Neuroscience, Royal Netherlands Academy of Arts and Sciences, Meibergdreef 47, 1105 BA Amsterdam, The Netherlands; 2 Cell Biology, Biophysics and Neurobiology, Department of Biology, Faculty of Science, University of Utrecht, Padualaan 8, 3584 CH Utrecht, The Netherlands; 3 Department of Anatomy and Neurosciences, Amsterdam University Medical Center, Vrij Universiteit, MS Center Amsterdam, De Boelelaan 1108, 1007 MB Amsterdam, The Netherlands; 4 Department of Neuroimmunology, Netherlands Institute for Neuroscience, Royal Netherlands Academy of Arts and Sciences, Meibergdreef 47, 1105 BA Amsterdam, The Netherlands; 5 Department of Immunology, University of Toronto, 1 King’s College Circle, Toronto, ON M5S 1A8 Canada; 6 Department of Psychiatry and Psychotherapy, Charité Universitätsmedizin Berlin, Campus Benjamin Franklin, Hindenburgdamm 30, 12203 Berlin, Germany; 7 Institut Für Neuroimmunologie Und Multiple Sklerose (INIMS), Universitätsklinikum Hamburg-Eppendorf, Hamburg, Germany

**Keywords:** Multiple sclerosis, Complement, Hippocampus, Synapses, Demyelination, Microglia

## Abstract

The complement system is implicated in synapse loss in the MS hippocampus, but the functional consequences of synapse loss remain poorly understood. Here, in post-mortem MS hippocampi with demyelination we find that deposits of the complement component C1q are enriched in the CA2 subfield, are linked to loss of inhibitory synapses and are significantly higher in MS patients with cognitive impairments compared to those with preserved cognitive functions. Using the cuprizone mouse model of demyelination, we corroborated that C1q deposits are highest within the demyelinated dorsal hippocampal CA2 pyramidal layer and co-localized with inhibitory synapses engulfed by microglia/macrophages. In agreement with the loss of inhibitory perisomatic synapses, we found that Schaffer collateral feedforward inhibition but not excitation was impaired in CA2 pyramidal neurons and accompanied by intrinsic changes and a reduced spike output. Finally, consistent with excitability deficits, we show that cuprizone-treated mice exhibit impaired encoding of social memories. Together, our findings identify CA2 as a critical circuit in demyelinated intrahippocampal lesions and memory dysfunctions in MS.

**Supplementary Information:**

The online version contains supplementary material available at 10.1007/s00401-021-02338-8.

## Introduction

Multiple sclerosis (MS) is an autoimmune demyelinating and neurodegenerative disease of the central nervous system (CNS). Patients living with MS experience major cognitive disabilities including memory impairment, attention deficits, and slowed sensory processing speed [9, 18], which occurs from the early stages of the disease [17]. Recent emerging insights have drawn particular attention to MS-related deficits in social cognition and facial emotion recognition as affected cognitive domains in MS, which can occur early in disease even in the absence of other cognitive problems [65] and may have distinct neuropathological substrates [10, 24]. There is substantial evidence that the hippocampus is critical for the consolidation and recollection of episodic memories, the temporal organization of events, and mapping of social space [73, 80]. Recent magnetic resonance imaging (MRI) studies have shown that structural and functional disconnections of the hippocampus from several brain networks can explain some of the clinical deficits experienced by MS patients including impaired memory and learning [46, 79] as well as depressive symptoms [22, 23]. In addition, post-mortem studies reported that the hippocampus of MS patients often shows extensive demyelination [15, 21, 71, 87], neuronal loss [58], synaptic alterations [14], neurotransmitter changes [35, 40] and inflammation [21, 28, 35].

The molecular and cellular basis of the MS-related hippocampal damage is, however, not fully understood. One leading hypothesis based on experimental [84, 90] and post-mortem studies [60, 90] indicates that the disconnection of temporal lobe networks in MS may be due to the loss of synapses via a “pruning” process. Over the past decade, several studies have identified proteins of the complement system as key components of the pruning process in development and learning [2, 33, 34, 74, 83, 86]. The complement system is traditionally known as a major arm of the innate immune system, required for optimal defense against pathogens and for the disposal of dead and dying cells [69]. The recently discovered role for complement in developmental synaptic pruning has been extensively investigated in the retinogeniculate system, where exuberant and overlapping synaptic connections are progressively segregated into eye-specific projections [75]. In this system, supernumerary synapses are targeted by the complement component 1q (C1q), opsonized by C3, and phagocytosed by microglia via complement receptor 3 (CR3) [74, 83]. In the rodent brain C1q has also been shown to play a role in shaping synaptic circuits in memory formation during adulthood [89], in ageing [82] and in neurodegeneration [33]. A more recent report showed that synaptic material is tagged by C3 (but not by C1q) and is engulfed by microglia in the retinogeniculate system of models of demyelination and in the visual thalamus of MS patients [90]. Our team previously showed that in the MS hippocampus C1q and C3d are deposited particularly in the CA2/3 region at synapses that localize within microglial processes and lysosomes, supporting a role for microglia in the elimination and degradation of synapses [51]. However, the nature of the targeted circuits, the mechanisms of synapse elimination and the functional consequences of synapse loss in the MS hippocampus are unknown.

Here, we first evaluated the extent of C1q depositions in CA2 versus CA3 regions of myelinated and demyelinated MS hippocampi, and related it to synaptic changes, MRI measures of brain atrophy, and the cognitive status of the patient. To further investigate the significance of C1q deposition and synaptic changes observed in the MS hippocampus, we made use of the cuprizone mouse model of demyelination. Because of the toxic nature of myelin loss, this model is particularly useful to investigate the role of C1q in synaptic changes independently of its role in antibody-mediated demyelination which occurs in other models such as experimental autoimmune encephalomyelitis. Using the cuprizone model, we first studied the extent and localization of C1q/C3 proteins in relation to synapses. Electrophysiological recordings were acquired, and social memory performance was tested to evaluate the consequences of subfield-specific synaptic changes in cuprizone-fed mice. Together, our findings identify the CA2 region of the hippocampus as a subfield that is highly susceptible to complement deposition and synaptic reorganization of the inhibitory circuit in MS. These changes may play a critical role in altering hippocampal information flow underlying cognitive deficits in the social domain.

## Materials and methods

### Human studies

#### Post-mortem hippocampal tissue collection

Post-mortem hippocampi of 55 MS donors and 12 non-neurological control (NNC) donors were obtained from the Netherlands Brain Bank (NBB; Amsterdam, the Netherlands). NBB autopsy procedures were approved by the Independent Review Board of the Amsterdam UMC, registered with US office of Human Research Protections. Written informed consent was obtained by the NBB for brain autopsy and for the use of material and clinical data for research purposes, in compliance with institutional and national ethical guidelines. Brains were removed according to a rapid (overall post-mortem delay (PMDS) of 6.7 ± 3.3 h, mean ± SD) autopsy protocol. Specimens were fixed in 10% buffered formalin and processed for embedding in paraffin. Paraffin-embedded hippocampi of all donors were used for the pathological study. Of these, hippocampi of 14 MS donors were selected for pathological-MRI correlation studies and hippocampi of 18 MS donors were selected for pathological-clinical correlation studies. Coronally cut hippocampi were selected to ensure accurate and systematic scoring of demyelination, C1q deposition and synaptic changes within the anatomical subfields of the hippocampus. MS cases and controls were matched for age; all numbers represent mean ± SD (MS myelinated [MS-M] donors: 65.2 ± 12.0 years; MS demyelinated [MS-DM] donors: 63.5 ± 15.3 years; NNC donors: 65.5 ± 10.1 years; one-way ANOVA *P* = 0.91) and PMD (MS-M donors: 7.4 ± 3.8 h; MS-D donors: 5.8 ± 2.3 h; NNC donors: 6.7 ± 3.1 h; one-way ANOVA *P* = 0.18). MS paraffin-embedded hippocampi used for immunostaining were from 29 donors with primary (PP) or secondary progressive (SP) disease and 26 donors with a progressive disease of undetermined type (PP/SP). In this study, PP, SP, and PP/SP donors were pooled and referred to as progressive MS. Detailed clinicopathological data of all donors are provided in Supplementary Table 1, online resource.

#### Magnetic resonance imaging (MRI)

The MRI protocol comprises both whole-brain in-situ MRI, and MRI of 10-mm thick coronal brain slices, which are cut at autopsy. A detailed description was previously published [76]. MR imaging was performed using 1.5 T Siemens Sonata and Avanto MRI scanners, depending on the availability at the time of autopsy, as described previously [64]. Briefly, the in-situ image acquisition protocol for volumetry of the hippocampus included a sagittal 3DT1-weighted imaging sequence (TR = 2700 ms, TE = 5.1 ms, TI = 950 ms, FA = 8, voxel size = 1.2 × 1.2 × 1.3 mm) and a sagittal 3D-FLAIR sequence (TR = 6500 ms, TE = 355 ms, TI = 2200 ms, voxel size = 1.2 × 1.2 × 1.3 mm). The 3DT1 images were used to measure whole hippocampus volumes corresponding with the hemisphere of the tissue samples extracted for the neuropathological assessment using the FreeSurfer image analysis suite version 5.3, which is documented and freely available for download online (http://surfer.nmr.mgh.harvard.edu/).

#### Evaluation of cognitive function

Inspecting the clinical data of all cases included in the MS post-mortem collection of the Netherlands Brain Bank (http://www.brainbank.nl/), we identified MS cases for which neuropsychological information was available. Using clinical chart information on cognitive status has proven successful in post-mortem research before [21]. By excluding any cases (1) without detailed information on cognition, (2) with a neuropsychological history (e.g. depression, character changes) and (3) that had any other non-MS pathology (e.g. vascular pathology), we were able to select high-quality post-mortem material from cognitively preserved (CP; *n* = 7) and cognitively impaired (CI; *n* = 7) MS patients. All included CI patients had memory problems that were often accompanied by disturbed linguistic capabilities. The demographic and clinical data of the selected CP and CI cases are summarized in Supplementary Table 1, online resource.

#### Hippocampal lesion classification

Hippocampal tissue sections were stained for proteolipid protein (PLP) and for the anti-human leukocyte antigen (HLA_DP-DQ-DR_). Because the distribution of HLA_DP-DQ-DR_–immunopositive microglial cells did not segregate with lesional areas, samples were scored for the presence of lesions according to their anatomical location and not lesion activity. Only intrahippocampal lesions were scored.

#### Immunohistochemistry

For immunohistochemistry, endogenous peroxidase activity was blocked by incubating the slides in methanol with 0.3% H_2_O_2_ for 20 min at room temperature (RT). Sections were washed in 1 × PBS (9 min) and put in a microwave on “High” power settings for 20 min in 10 mM Tris/1 mM Ethylenediaminetetraacetic acid (EDTA) buffer pH.9 (Supplementary Table 2, online resource). Sections were rinsed in 1 × PBS, outlined with a hydrophobic pen, washed in 1 × PBS and PBST (3 min). The sections were then blocked with normal goat serum in PBST (1:1) for 30 min at RT before being incubated with the relevant primary antibody (Supplementary Table 2, online resource) diluted in Normal Antibody Diluent (Immunologic, Duiven, The Netherlands) for 1 h at RT and then overnight at 4 °C. The next day, slides were rinsed in PBST (9 min) and incubated with Post Antibody Blocking BrightVision Solution 1 (diluted 1:1 in PBST, ImmunoLogic) for 15 min at RT. They are then washed in 1 × PBS and incubated with BrightVision Poly-HRP-Anti Ms/Rb/Rt IgG Biotin-free Solution 2 (diluted 1:1 in PBST, ImmunoLogic) for 30 min at RT. The immunostaining was visualised using 3,3’-Diaminobenzidine (DAB, Sigma-Aldrich) for 4 min at RT. The sections were counterstained with hematoxylin. Sections were then dehydrated in a series (50%, 70%, 100%, 100%) of ethanol and xylene (3 min). The slides were mounted using Entellan medium. All stained images were scanned using an Axio Imager Z1, Zeiss microscope connected to a digital camera (AxioCam 506 mono, Zeiss) and imaged with Zen pro 2.0 imaging software (Zeiss).

#### Immunofluorescence staining

For the fluorescent immunostaining of pre- and postsynaptic elements, sections were pretreated with microwave antigen retrieval as described above. Primary antibodies against the presynaptic elements vesicular glutamate transporter 1 (vGLUT1) or vesicular GABA transporter (vGAT) (see Supplementary Table 2, online resource) were diluted in normal antibody diluent (Immunologic, Duiven, the Netherlands) and incubated for 3 h at RT followed by overnight incubation at 4 °C. The next day, sections were washed in PBS and incubated in primary antibodies against the postsynaptic elements postsynaptic domain 95 (PSD95) or Gephyrin (see Supplementary Table 2, online resource) diluted in normal antibody diluent for 4 h at RT followed by 2 overnights incubation at 4 °C. Two days later, sections were washed in PBS and incubated in polyclonal IgG donkey anti-guinea pig Alexa488-conjugated (Jackson, A-S155) and the polyclonal IgG donkey anti-rabbit Alexa546-conjugated (Invitrogen, A-S154) secondary antibodies or the polyclonal IgG donkey anti-chicken Alexa488-conjugated (Jackson, A-S153) and the polyclonal IgG donkey anti-mouse Alexa546-conjugated (Molecular probes, A-S032) secondary antibodies diluted 1:200 in PBS supplemented with 3% donkey serum with 0.1% triton for 3 h at RT. After washing in PBS, sections were incubated with 40,6-diamidino-2-phenylindole (DAPI; Vector Laboratories) to visualize the nuclei, incubated in Sudan Black B for 5 min at RT. After washing in 70% ethanol and sqH_2_O, the slides were air-dried and mounted in an aqueous mounting medium. For the fluorescent immunostaining of C1q, sections were pretreated with microwave antigen retrieval as described above. Primary antibody against C1q (see Supplementary Table 2, online resource) was diluted in normal antibody diluent and incubated for 3 h at RT followed by overnight incubation at 4 °C. The next day, sections were washed in PBS and incubated in polyclonal IgG goat anti-mouse Alexa488-conjugated (Thermo Fisher, ab235454) secondary antibody diluted 1:200 in PBS supplemented with 3% donkey serum with 0.1% triton for 3 h at RT. Following counterstaining with DAPI, sections were washed and air dried as described above before they were imaged. Using the appropriate filters, the immunofluorescence signal was visualized with an Axio Imager Z1, Zeiss microscope connected to a digital camera (AxioCam 506 mono, Zeiss) and imaged with Zen pro 2.0 imaging software (Zeiss). To control for antibody specificity, tissue sections were stained according to the IF or IHC protocols described above except for the primary antibody incubation step, which was omitted.

#### Quantification of immunohistochemistry

Formalin-fixed paraffin-embedded tissue blocks were cut into 7 µm sections on a microtome (ThermoScientific HM 325), mounted onto Superfrost Plus glass slides, and dried overnight at 37 °C. Sections were deparaffinized in xylene (2 × 5 min), rehydrated through a series (100%, 70%, 50%) of ethanol and sqH_2_O (3 min). For the CA2 and CA3 subfields 3 randomly selected nonoverlapping digital images were captured for quantification. Therefore, for each immunostaining, a total of 72 images (3 images × 2 subfields × 12 donors) of NNCs, 186 images (3 images × 2 subfields × 31 donors) of MS-M hippocampi, and 144 images (3 images × 2 subfields × 24 donors) of MS-DM hippocampi were captured at × 20 magnification and analysed. Quantitative analysis of immunostaining was performed on the region of interest (ROI) using the ‘measurement’ function of ImageJ 1.15 s (National Institutes of Health). Briefly, the RGB images were separated into single color channels using the color deconvolution plugin in Image J. The single-color channel for each staining was subjected to thresholding to create a mask that captures the specific staining. The threshold was saved and applied to all images in the same staining group. The area fraction measurement was applied to each ROI to quantify the percentage of thresholded staining. The amount of staining is expressed as a percentage of the immunoreactive area over the total area assessed. Quantitative analysis of the number of NeuN^+^ cells was performed using the ‘analyse particle’ function of ImageJ 1.15 s. Briefly, the RGB image was subjected to the color thresholding to create a mask that captures the specific staining. The threshold was saved and applied to all images in the same staining group. The ‘analyze particle’ function was applied to each ROI to quantify the number of selected “particles”. Quantitative analysis of the number of PV^+^ cells was performed by manual counting. For the quantification of the area of the ROI, the area measurement function was used after the images were calibrated. The NeuN^+^ or PV^+^ cells were expressed as a number of cells per mm^2^ of CA2 or CA3.

### Animal studies

All animal experiments were performed in compliance with the European Communities Council Directive 2010/63/EU effective from 1 January 2013. The experiments were evaluated by the KNAW Animal Ethics Committee (DEC) and Central Authority for Scientific Procedures on Animals (CCD, license AVD8010020172426). The specific experimental designs were evaluated and monitored by the Animal Welfare Body (IvD, protocols NIN18.21.01, NIN19.21.06 and NIN19.21.07). Male C57BL/6 mice (Janvier Labs, Saint-Berthevin Cedex, France) were kept on a 12:12 h light–dark cycle (lights on at 07.00 am, lights off at 19.00 pm) with ad libitum food and water. Demyelination was induced by cuprizone feeding [27]. From the age of 5–6 weeks and a bodyweight > 20 g (on average 21.6 g, range: 20.5–22.8 g), mice were fed with 0.2% cuprizone supplemented to the powder food, freshly prepared every second day for a period between 2 and 9 weeks as indicated in the text. The associated weight loss with cuprizone treatment was assessed every second day and monitored in consultation with the IvD.

#### Hippocampus slice preparation and electrophysiological recordings

Mice received a terminal dose of Nembutal (5 mg kg^–1^) and were transcardially perfused with ice-cold artificial cerebrospinal fluid (ACSF) consisting of (in mM): 87.0 NaCl, 25.0 NaHCO_3,_ 2.5 KCl, 25.0 NaH_2_PO_4_, 75.0 sucrose, 25.0 glucose, 0.5 CaCl_2_ and 7.0 MgCl_2_ (oxygenated with 5% CO_2_–95% O_2_, pH 7.4). After decapitation, the brain was quickly removed from the skull and the hippocampus was isolated from the inside of the cortical mantle in an ice-cold (0 to + 4 °C) dissecting solution. The isolated hippocampus was placed in the groove of an agar block with the anterior part facing upward. Transverse hippocampal slices (400 µm) were cut starting at the dorsal site of the hippocampus using a Vibratome (1200VT, Leica Microsystems). Slices were allowed a recovery period of 30 min at 35 °C and were subsequently stored at room temperature in a solution containing 125 NaCl, 3 KCl, 25 glucose, 25 NaHCO_3_, 1.25 Na_2_H_2_PO_4_, 1 CaCl_2_, 6 MgCl_2_, 1 kynurenic acid (95% O_2_ and 5% CO_2_, pH 7.4). For patch-clamp recordings, slices were transferred to an upright microscope (BX51WI, Olympus Nederland) equipped with oblique illumination optics (WI-OBCD; numerical aperture, 0.8). CA2 pyramidal cells located deep in the slice were visualized using 40 × water-immersion objectives (Olympus) and oblique LED illumination optics (850 nm) based on the curvature of the pyramidal layer, the typical large soma, and triangle shape. Some neurons showed a proximal bifurcation in the main apical dendrite. The microscope bath was perfused with oxygenated (95% O_2_, 5% CO_2_) ACSF consisting of the following (in mM): 125 NaCl, 3 KCl, 25 D-glucose, 25 NaHCO_3_, 1.25 Na_2_H_2_PO_4_, 2 CaCl_2_, and 1 MgCl_2_.

Patch pipettes were pulled from borosilicate glass (Harvard Apparatus, Edenbridge, Kent, UK) to an open tip of 3–6 MΩ resistance. For all current-clamp recordings, the intracellular solution contained (in mM): 130 K-Gluconate, 10 KCl, 4 Mg-ATP, 0.3 Na_2_-GTP, 10 HEPES and 10 Na_2_-phosphocreatine (pH 7.25 adjusted with KOH, 280 mOsmol kg^−1^). The liquid junction potential difference of –13.5 mV was corrected in all recordings. All voltage recordings were analogue low-pass filtered at 10 kHz (Bessel), recorded using DAGAN BVC 700 amplifiers, and digitally sampled at 100 kHz using an ITC-18 A-D converter (HEKA Elektronik Dr. Schulze GmbH, Germany). Bridge-balance and pipette or stray capacitances were fully compensated based on small current injections leading to minimal voltage errors. All data acquisition and analyses were performed with Axograph X (v.1.7.0, NSW, Australia, https://www.axograph.com/). The recording temperature was 34 ± 1 °C. Only cells with a stable bridge-balance (< 25 MΩ) and resting membrane potential throughout the recording session were included in the analyses.

#### Morphological analysis and pyramidal cell identification

For single-cell biocytin-labelling, recorded neurons were filled with 5 mg ml^–1^ biocytin for at least 30 min and fixed overnight in 4% PFA. Streptavidin biotin-binding protein (Streptavidin Alexa 488, 1:500, Invitrogen) was diluted in 5% BSA with 5% NGS and 0.3% Triton-X overnight at 4 °C. To identify the CA2 region, primary antibody rabbit anti-PCP4 (1:250 Sigma Aldrich, HPA005792) or mouse anti-RGS14 (1:500, Neuromab) were added to an overnight incubation mix. Secondary antibodies were Alexa 633 goat anti-rabbit (1:500; Invitrogen) or Alexa 633 goat anti-mouse (1:500; Invitrogen). Brain slices were mounted on glass slides and cover slipped with Vectashield H1000 fluorescent mounting medium (Vector Laboratories, Peterborough, UK) and sealed. Sections were imaged using a confocal laser-scanning microscope (SP8, DM6000 CFS; acquisition software, Leica Application Suite AF v3.2.1.9702) with 40 × oil-immersion objectives and 1 × digital zoom with step size of 0.5 µm. Alexa 488 and Alexa 633 were imaged using 488 and 633 excitation wavelengths, respectively. Confocal *z*-stacks were imported into Neurolucida 360 software (v2020, MBF Bioscience) for reconstruction using the interactive user-guided trace with the Directional Kernels method. Axon, basal and apical dendrite segments, as well as the spine type and densities were analyzed using Neurolucida Explorer (MBF Bioscience). Tracing was performed blind for the experimental groups.

#### Immunohistochemistry and synapse staining

Mice were anaesthetised with Nembutal (5 mg kg^–1^), the brain rapidly removed and immersion-fixed with 4% PFA overnight at room temperature. The fixed brains were briefly rinsed in PBS (Phosphate Buffer Solution) before sunken in 30% sucrose/PBS solution at 4 °C, frozen with dry ice and stored at –80 °C. One day before the experiment the frozen brains were moved to –20 °C and stored overnight. On the day of the experiment, 14 µm sagittal or coronal sections were produced with a freezing-sliding microtome and stored in PBS at 4 °C. Free-floating sections were permeabilized at RT with 10% normal goat serum in 0.3% Triton X-100 in PBS for 2 h, followed by primary antibody incubation overnight at 4 °C. Primary antibodies used, dilution, and sources are provided in Supplementary Table 2, online resource. After rinsing 3 × in PBS for 15 min, sections were incubated with secondary antibodies (1:500) in PBS with 3% goat serum for 2 h at room temperature. After rinsing 3 × in PBS for 15 min, sections were mounted on glass slides, using vectashield containing DAPI (Vector labs H-1000). Fluorescence signals were imaged with a Leica TCS SP5 II (DMI6000 CFS; acquisition software Leica Application Suite AF v. 2.6.3.8173) or SP8 confocal laser-scanning microscope (DM6000 CFS; acquisition software, Leica Application Suite AF v3.2.1.9702, Leica Microsystems GmbH). Confocal images used for the intensity analysis were acquired at 1096 × 1096 pixels (2.0 or 3.0 μm *z*-step) using a 10 × objective. Density of puncta were acquired at 1096 × 1096 pixels (2.0 or 3.0 μm *z*-step) using a 40 × or 63 × oil-immersion objectives (0.75–1.0 digital zoom). To avoid bleed-through between emission wavelengths, automated sequential acquisition of multiple channels was used, and images saved as uncompressed LIF format. Immunofluorescence was quantitatively analyzed in FIJI using either a mask and averaging signal intensity (arbitrary units) or as a percentage of the immunoreactive area over the total area assessed a described above for the human post-mortem tissue. Puncta analysis was performed by quantifying the number of immunopositive puncta over the total area assessed (expressed in μm^2^). Surface reconstructions of vGAT^+^ /Iba-1^+^ /LAMP1^+^ cells were made with Imaris (v. 9.6, Bitplane AG, Zurich, Switzerland).

#### Electron microscopy

Tissue for electron microscopy was obtained from adult mice that were transcardially perfused and postfixed with freshly prepared 2% PFA and 2.5% glutaraldehyde in a 0.1 M phosphate buffer (PB) pH 7.4. All steps were done at room temperature unless stated otherwise. After subsequent washes in PB, tissues were cryo-protected through a gradient of 10%, 20% and 30% sucrose in PB and frozen on aluminium boats on dry ice. Coronal sections of 40 μm containing the hippocampus were obtained using a freezing microtome. Frozen coronal sections of the hippocampus were washed in PB, slices were blocked 2 h with 5% normal goat serum in PB and incubated overnight with rabbit-α-C1q antibody (1:1000 in blocking solution) while shaking. Slices were washed in PB, incubated for 2 h with a horseradish peroxidase coupled rabbit secondary antibody, washed in PB, pre-incubated for 20 min with 0.05% 3,3′-diaminobenzidine (DAB) in PB and incubated for 5’ with DAB and 0.03% H_2_O_2_ for visualization. The DAB reaction product was then intensified using the gold-substituted silver peroxidase method as previously described [85]. Briefly, slices were rinsed in 2% sodium acetate buffer and incubated for 2 h in 10% sodium thioglycolate on a shaker. After multiple washes with the acetate buffer, slices were incubated for 6 min with silver solution, consisting of 2.5% sodium carbonate, 0.1% ammonium nitrate, 0.1% silver nitrate, 0.5% tungstenosilic acid and 0.07% formalin. Following washes with acetate buffer, slices were incubated with 0.05% gold chloride for 20 min, rinsed with acetate buffer, and incubated with 3% sodium thiosulfate for 5 min. After rinsing with acetate buffer, slices were rinsed several times with 0.1 M sodium cacodylate buffer (pH 7.4) and post-fixed for 20 min in 1% osmium tetroxide supplemented with 1.5% ferricyanide in cacodylate buffer. Subsequently, the tissue was dehydrated using a gradient of 30%, 50%, 70%, 80%, 90% and 100% ethanol followed by acetone. After incubating for 30 min in a 1:1 mixture of acetone with epoxy, slices were incubated for 30 min in pure epoxy and left at 65 °C overnight to harden. With an Ultracut UCT ultrathin 70 nm sections were made and collected on electron microscopy grids with a formvar film. Contrasting of the tissue was achieved by incubation with 0.5% uranyl acetate for 4 min, followed by extensive washing with milliQ and drying to the air, and subsequent incubation with lead citrate for 2 min. Ultrathin sections were examined with a FEI Tecnai G12 electron microscope (FEI, Europe NanoPort, Eindhoven, the Netherlands) and images obtained with a Veleta camera, acquired as 16-bits TIF files. Images were saved in tiff format and analyzed using Fiji (ImageJ). We examined > 100 sections from 3 mice/group.

#### Anti-mouse MOG IgG ELISA

Serum was collected from animals at various timepoints following transfer to cuprizone diet and were kept frozen at –20 °C until required. Samples were assayed using Anaspec SensoLyte ® Anti-Mouse MOG_(1–125)_ IgG Quantitative ELISA kits (AS-55156). Briefly, 96-well plates precoated with recombinant mouse MOG_(1–125)_ protein were incubated with 50 mL of the appropriate samples or standard with gentle shaking at RT for 1 h. Each sample was diluted in sample buffer at 1:100 and subject to 1:4 serial dilutions up to 1:6400. Each sample was plated in duplicate on the precoated/preblocked plate. Following sample incubation, samples were washed 5 times with wash buffer and incubated with anti-mouse IgG-HRP (1:2000 dilution) with gentle shaking at RT for 1 h. Following incubation with a secondary antibody, the plate was washed 5 times and 100 mL of TMB was added to detect the level of anti-MOG IgG via optical density at 450 nm using a spectrophotometer. Serum from hMOG-immunized EAE mice at the chronic phase of disease was used as a positive control for this assay and was assayed at a starting dilution of 1:100 subjected to 1:4 serial dilutions up to 1:1,638,400.

#### Behavioral tests

The *five-trial social memory test* was based on the design from Hitti & Siegelbaum [31]. All mice (*n* = 11 control and 11 cuprizone treated mice, 0.2% for 7 weeks) were maintained group-housed (3–4 mice/cage) before the test and the sequence of testing was determined randomly. Social memory tests were performed between 08.00 am and 03.00 pm. For the test, the subject mouse was transferred to the experimental room and allowed to familiarize with the cage for 15 min with the lid closed. After 15 min, the lid was removed, and the webcam recording started (~ 30 Hz frame rate). At this point, the subject mouse was exposed to a novel male mouse for the duration of 1 min (trial 1). The novel mouse was removed for 10 min. Subsequently, the same procedure was repeated three more times (i.e. subject mouse exposed to the familiar mouse, trials 2, 3 and 4). In trial 5, an unfamiliar mouse was introduced to measure dishabituation. The behaviors of the subject mouse were analysed off-line. The behavioural scoring included the duration of anogenital sniffing, approaching behavior, social interaction, aggressive interaction or no interaction. The occurrence and durations of these distinct behaviors were measured by two different researchers, both blinded to the animal identities until the data were analysed and plotted.

For automated *discrimination learning* experiments (Sylics Bioinformatics, Amsterdam, The Netherlands) we used PhenoTyper cages (model 3000, Noldus Information Technology). The system is an automated home cage in which behavior is tracked by a video. The cage is equipped with a drinking bottle and a triangular-shaped shelter with two entrances in one corner. In the opposite corner, an aluminum tube of an automated food reward dispenser protruded into the cage. Mice (*n* = 9 control and 13 cuprizone-treated mice, 0.2% for 6 weeks) had ad libitum access to drinking water but needed to engage for food reward in the Cognition Wall discrimination test. The wall contained three entrances and when they passed through the left entrance, they automatically obtained a food pellet (Dustless Precision Pellets, 14 mg, Bio-Serve). The rate at which a mouse gains a relative preference for the rewarded entrance is used as a measure of discrimination learning. Mice were single housed for 1 week to accommodate to the 16 h periods in which they were housed in the PhenoTyper cages and the experiment started 3 h before lights-off (04.00 pm). C57BL/6 J mice require typically around 100 food rewards/per day to maintain body weight. We analyzed the total number of entries needed to reach a criterion of 70% to 90% correct, computed as a moving window over the last 30 entries to assess learning in the task. Since this performance measurement uses the fraction of correct over incorrect entries in the last 30 entries rather than the total number of entries or latency to reach criterion, this measurement is not likely to be influenced by general differences in activity between genotypes or groups. Hence, mice cannot achieve the learning criterion by only showing increased motor activity and making more entries.

### Statistical analysis

**Fig. 1:**
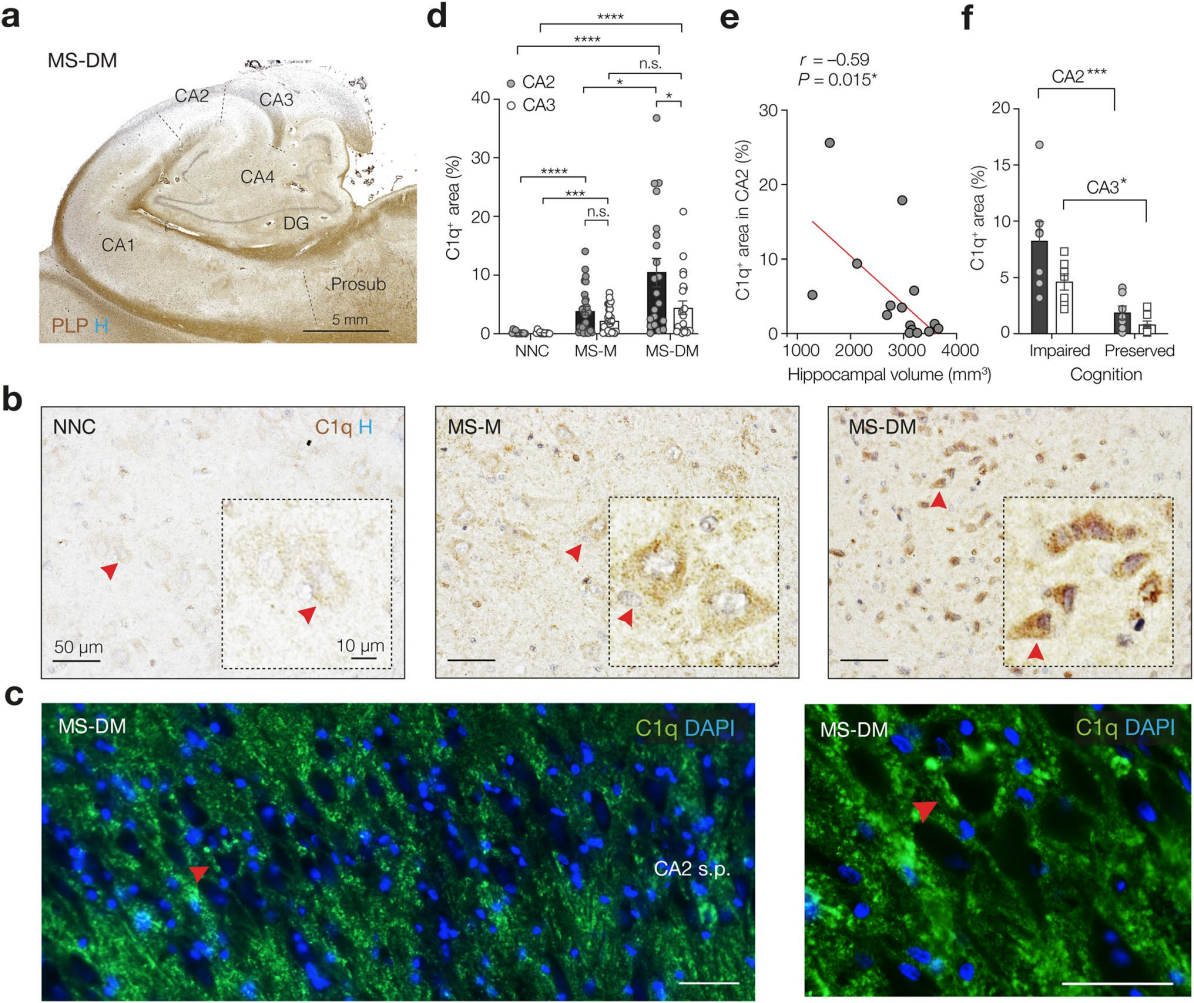
The multiple sclerosis hippocampus shows demyelination-dependent increase of C1q-immunoreactive area in the CA2. **a** Coronal view of the hippocampus of an MS case showing hippocampal demyelination (MS-DM) in CA2 and CA3 assessed by proteolipid protein (PLP, brown) and hematoxylin (blue) staining. **b** Higher magnification of the CA2 subfield of hippocampi from a non-neurological control case (NNC), an MS case with myelinated hippocampus (MS-M) and an MS-DM case. Red arrows indicate the location of the insets showing individual CA2 neurons at higher magnification. Note the C1q immunoreactivity (brown) in the soma of hippocampal neurons as well as the C1q^+^ punctate staining pattern throughout the tissue, particularly in MS-M and MS-DM hippocampi. Decentrated neuronal nuclei are visible in the inset of MS-DM. Blue, Hematoxylin. Red arrows indicated cells magnified in the insets.**c** Higher magnification of the CA2 subfield from an MS case with demyelinated hippocampus (MS-DM) stained by immunofluorescence for C1q (green) and DAPI (blue). Note the C1q^+^ punctate staining pattern in the perisomatic areas of neurons (red arrow) in the CA2 stratus pyramidalis (s.p.) Scale bars, 50 µm. **d** Quantification of C1q^+^ area in post-mortem hippocampal CA2 and CA3 subfields, showing a significant increase in MS cases compared to controls (Kruskal–Wallis adjusted *P* < 0.0001, NNC *n* = 12, MS-M *n* = 30, MS-DM *n* = 21). The C1q^+^ area was significantly higher in MS-DM CA2 compared to MS-M CA2 and compared to MS-DM CA3 (*adjusted *P* = 0.03 and *adjusted *P* = 0.04 respectively). Bars represent the mean ± SEM; Grey circles and open squares represent individual hippocampi for CA2 and CA3 areas, respectively. **e** Spearman’s correlation analysis reveals a significant negative correlation (*r*) between the C1q^+^ area in CA2 and hippocampal volume as determined by post-mortem MRI in a subcohort of MS cases (two-tailed exact *P* = 0.0145, *n* = 17 hippocampi). **f** Quantification of C1q^+^ area in MS donors with impaired cognitive/memory function compared to donors with preserved cognitive/memory function reveals a higher percentage of C1q immunoreactive area in MS donors with impaired cognitive/memory function compared to donors with preserved cognitive/memory function (two-way ANOVA cognition effect *F*_1, 24_ = 26.44, ***P < 0.0001), and higher levels in CA2 (two-way ANOVA subfield effect *F*_1, 24_ = 5.68, *P* = 0.0254, *n* = 7 biological replicates for all groups). C1q was increased in cognitively impaired MS patients, both in CA2 and CA3 (Šidák’s multiple comparison test CA2, *t* = 4.55, *df* = 24, ***P < 0.0001 and CA3, *t* = 2.73, *df* = 24, *P < 0.01, respectively). Bars represent the mean ± SEM; Circles and squares represent individual hippocampi for CA2 and CA3 areas, respectively

All tests were performed using GraphPad Prism software (v. 9.1.1, GraphPad Software Inc, San Diego, CA, USA). Sample sizes for the animal experiments and electrophysiological recordings were determined by performing power tests with a type II error set to 0.8. The type of variability of distributions was assessed by Shapiro–Wilk normality test. The non-normally distributed data were analysed with non-parametric Mann–Whitney test if two groups were compared or with the non-parametric Kruskal–Wallis test followed by Dunn’s correction for multiple comparisons if > 2 groups were compared or followed by a false discovery rate approach using the two-stage linear step-up procedure of Benjamini, Krieger and Yekutieli (Fig. 1d). Correlation analyses of non-normally distributed data were performed by Spearman correlation coefficient. If data were normally distributed, data groups were analysed by either ordinary two-way or repeated measures (RM) parametric analysis of variance (ANOVA) followed by post-hoc analyses with Šidák’s, Holm-Šidák or Bonferroni’s multiple comparisons tests. For all tests, the null hypothesis was rejected with *P* < 0.05 at a 95% confidence interval.

### Data availability

All raw data supporting the findings of this study are available from the corresponding authors upon reasonable request.

## Results

### CA2 enrichment of C1q deposits in the atrophic demyelinated MS hippocampus

To test for demyelination-dependent or -independent changes in C1q, the immunohistochemical analyses conducted in this study included both myelinated and demyelinated MS hippocampi. Furthermore, because C1q expression in the CNS increases with normal ageing [82], we age-matched the donors to control for age-dependent changes in our samples. Using a collection of post-mortem hippocampal tissue from 55 MS cases and 12 non-neurological controls (NNC), we first performed immunostaining for the PLP marker of myelin and identified 31 cases with myelinated, lesion-free, MS hippocampus (MS myelinated, MS-M) and 24 MS cases with partly or completely demyelinated hippocampus (MS demyelinated, MS-DM) (Fig. 1a). MS cases with hippocampal demyelination and those without hippocampal demyelination did not differ in terms of disease duration (measured from the time of first symptoms to the time of death) and age at death (Supplementary Fig. 1, online resource). Consistent with previous work [51], the hippocampi from NNCs showed no sign of demyelination. In addition, and in line with previous reports [21, 40], the MS samples showed only a slightly increased HLA-DP-DQ-DR staining, suggesting enhanced microglial reactivity generally restricted to hippocampal areas with preserved PLP myelin staining (data not shown).

Using immunohistochemistry, western blot and in situ hybridization on post-mortem MS hippocampi—including those used in this study—our previously published work indicated that the amount of C1q immunoreactivity in the MS hippocampus was high in CA2/3 compared to other hippocampal subfields, including CA1 and subiculum, and localized at neuronal soma as well as synapses [51], but in that study, we did not distinguish between CA2 and CA3 subfields. Since it is becoming increasingly clear that the CA2 hippocampal subfield has a cytoarchitecture, connectivity, gene expression, and neurochemistry functionally distinct from CA3 (and CA1) [13, 31, 38, 57], we aimed to examine whether demyelination may have subfield-specific alterations. Given the lack of molecular markers that can differentiate between the CA2 vs CA3 subfields in humans, the boundaries between these two subfields were determined in situ based on cytoarchitectural criteria such as the higher cell packing density and larger CA2 pyramidal neurons somata compared to CA3 [91]. Using colorimetric immunohistochemistry and immunofluorescence, we confirmed the previously observed [51] punctate staining pattern of C1q in the somata of hippocampal neurons (Fig. 1b), some of which had a dystrophic appearance with decentrated nuclei suggestive of neuronal injury (inset, Fig. 1b), as well as in the perisomatic area of neurons. The latter was more obvious when visualized with high contrast by immunofluorescence microscopy especially in the stratum pyramidalis of CA2 in MS-DM cases (Fig. 1c). Comparative analysis of the percentage of area covered by the C1q immunostaining showed that in the CA2 region of MS-DM hippocampi the amount of C1q deposition was on average ~ tenfold higher compared to NNCs and ~ threefold higher compared to MS-M (adjusted *P* < 0.0001 for both, Fig. 1d) but not within CA3 (Fig. 1d). Because the percentage of C1q^+^ area was highest in CA2 compared to CA3 in the demyelinated MS hippocampus (adjusted *P* = 0.044) we next asked whether the extent of C1q immunoreactivity in CA2 could be linked to hippocampal atrophy. Correlation analyses between the percentage of C1q^+^ area and volumetric changes of the hippocampus as measured by post-mortem MRI revealed a significant correlation between the extent of C1q immunoreactivity and hippocampal volume (*P* = 0.015, Fig. 1e), demonstrating an association between C1q in CA2 and hippocampal atrophy in progressive MS donors. Furthermore, since the hippocampus is of critical importance for spatial and emotional memory, we next asked whether there is a link between the extent of C1q immunoreactivity in CA2 and cognitive functions. Comparison of the immunofluorescence density of C1q in MS cases with or without documented impairment of cognitive function (based on available clinical records) showed that MS cases with impaired cognitive function had a significantly and 4.3-fold higher amount of C1q deposits in CA2 than those patients without evidence of cognitive problems (Sidak’s multiple comparison test *P* < 0.001, Fig. 1f). While the difference was also detectable in CA3 (Sidak’s test *P* < 0.05, Fig. 1f) the C1q expression was substantially higher within the CA2 region (two-way ANOVA, subfield effect *P* < 0.0254). Together with the significantly higher expression in the larger data set (Fig. 1d) these results indicate that the CA2 region shows an increased sensitivity to complement activation in MS.

### Loss of GABAergic and gain of glutamatergic synaptic elements in the CA2 subfield of the MS hippocampus

**Fig. 2:**
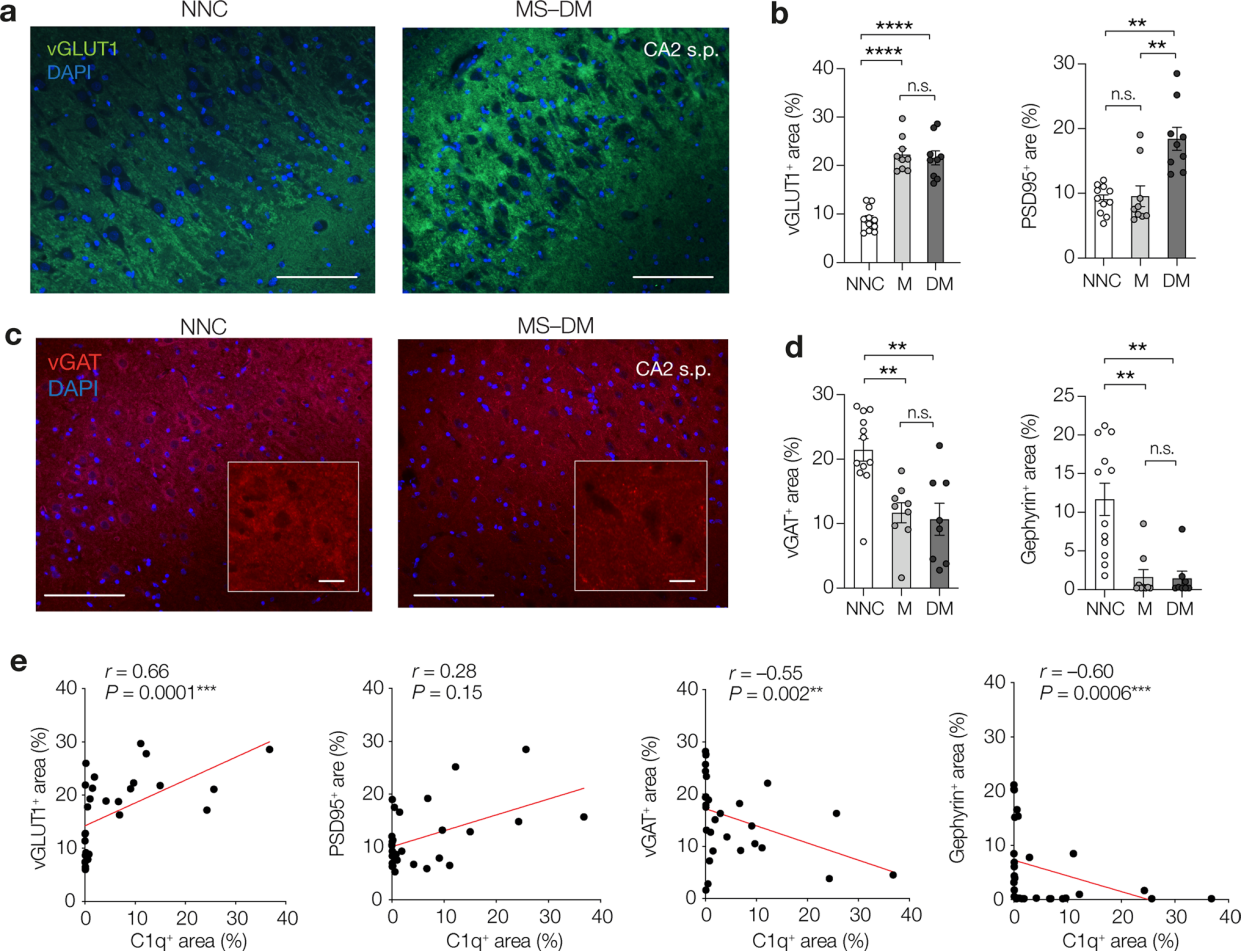
Changes in synaptic elements associate with the extent of C1q immunoreactivity in the MS CA2 region. **a** Immunofluorescence staining for vGLUT1 (in green) and DAPI (in blue) in CA2 stratum pyramidale (s.p.) of a non-neurological control (NNC) case and an MS case with demyelinated hippocampus (MS-DM). Scale bar, 50 µm. **b** Quantification of vGLUT1^+^ area and PSD95^+^ area in CA2 of non-neurological control (NNC) cases and MS cases with myelinated (MS-M) or demyelinated (MS-DM) hippocampi, showing increased vGLUT1^+^ area (One-way ANOVA *F*_2, 26_ = 47.88, *P* < 0.0001, Šidák’s multiple comparison tests, MS-M versus NCC, *t* = 8.55, ****P < 0.0001; MS-DM versus NCC, *t* = 8.10, ****P < 0.00001, MS-M versus MS-DM *t* = 0.43, *P* = 0.96) and increased PSD95^+^ area in demyelinated MS cases (Kruskal–Wallis test = 14.26, *P* = 0.0008, Dunn’s multiple comparison tests, MS-M versus NCC, *z* = 0.18, *P* > 0.99; MS-DM versus NCC, *z* = 3.29, **P = 0.0031 and MS-M versus MS-DM *z* = 3.31, **P = 0.0028). **c** Immunofluorescence staining for vGAT (in red) and DAPI (in blue) in CA2 stratum pyramidale (s.p.) of a non-neurological control (NNC) case and an MS case with the demyelinated hippocampus (MS-DM). Scale bar, 50 µm. Inset shows loss of perisomatic clustering of vGAT^+^ signal in MS-DM. Scale bar, 10 µm. **d** Quantification of vGAT^+^ area and gephyrin^+^ area in CA2 reveals MS decreased vGAT^+^ area (One-way ANOVA *F*_2, 26_ = 10.51, *P* = 0.0005, followed by Šidák’s multiple comparison tests; MS-M versus NNC, *t* = 3.72, **P = 0.0028; MS-DM versus NNC, *t* = 3.97, **P = 0.0015, MS-DM versus MS-M, *t* = 0.35, *P* = 0.98, *df* = 26 for all) as well as decreased gephyrin^+^ area in MS (Kruskal–Wallis test = 16.07, *P* = 0.0003, Dunn’s multiple comparison tests, MS-M versus NCC, *z* = 3.37, **P = 0.0023; MS-DM versus NCC, *z* = 3.37, **P = 0.0022 and MS-M versus MS-DM *z* = 0.11, *P* > 0.99). **e** Correlation analyses between the C1q^+^ area and synapse markers in CA2 of MS cases (M and DM, *n* = 29). Spearman’s correlation analyses show a significant positive correlation between the C1q^+^ area and the vGLUT1^+^ area whereas it shows significant negative correlations between the C1q^+^ area and inhibitory markers vGATand gephyrin. Two-tailed *P* values and correlation coefficients (*r*) are indicated in the figure panels

While a common finding from our previous studies [51] and others [14, 58], is that synapses are lost in the MS hippocampus, which synapses are selectively changed within the CA2 hippocampal subfield is not well understood. We performed immunofluorescence staining for the presynaptic vesicular glutamate transporters 1 (vGLUT1) and a postsynaptic element of excitatory synapses the postsynaptic domain 95 (PSD95), as well as a presynaptic marker for gamma-aminobutyric acid (GABA)ergic synapses, the vesicular GABA transporter (vGAT), and a postsynaptic element of inhibitory synapses (gephyrin). Quantification analysis of presynaptic elements in CA2 showed that compared to NNCs the amount of vGLUT1^+^ area was increased in MS tissue by ~ 2.5-fold (*P* < 0.0001) while the amount of vGAT^+^ area in MS hippocampi was decreased 2.5-fold (*P* = 0.0005) (Fig. 2a–d). Similar changes were observed in the CA2 of MS-M cases (vGLUT^+^ area, ~ threefold increase in MS-M vs NNCs, *P* < 0.0001; vGAT^+^ area, 2.5-fold decrease in MS-M vs NNCs, *P* = 0.0028, Fig. 2a–d). Furthermore, quantification analysis of postsynaptic elements in CA2 showed that compared to NNCs, the amount of PSD95^+^ area was increased by twofold in the demyelinated MS hippocampus (*P* = 0.0031) while, in striking contrast, the amount of gephyrin^+^ area decreased in MS-DM hippocampi (*P* = 0.0022, Fig. 2). Interestingly, while similar changes in gephyrin^+^ postsynaptic elements were observed in the CA2 of MS-M cases (6.6-fold decrease in MS-M vs NNCs, *P* = 0.0023), no changes were observed in PSD95^+^ postsynaptic elements of the CA2 of MS-M cases (*P* > 0.99, Fig. 2). These findings indicate a gain of excitatory postsynaptic elements and a concomitant loss of inhibitory postsynaptic elements in the CA2 subfield of the MS hippocampus. Furthermore, they suggest that changes in inhibitory but not excitatory CA2 postsynaptic elements may precede demyelination in MS.

Since neuronal loss has been documented in the MS hippocampus [58], we next asked whether changes in synaptic elements could be the consequence of neuronal loss. To answer this question, the number of neurons per hippocampal area was quantified based on immunohistochemistry for the neuronal nuclei (NeuN) marker of neurons in CA2 and CA3 of MS hippocampi compared to controls. In line with previous literature [58], we found a significant ~ 30% loss of neurons in both subregions compared to controls (Supplementary Fig. 2, online resource). Furthermore, since the loss of inhibitory interneurons has been documented in the MS cortex [92], we next asked whether changes in inhibitory synaptic elements could be the consequence of loss of inhibitory interneurons in the MS hippocampus. To answer this question, the number of inhibitory parvalbumin (PV)^+^ interneurons per hippocampal area was quantified based on immunohistochemistry for the PV marker of neurons in CA2 and CA3 of MS hippocampi compared to controls. We found that the density of PV^+^ neurons in CA2 was decreased by ~ 2.2-fold between NNCs and MS-M (*P* = 0.002) as well as by ~ 10.8-fold between NNCs and MS-DM (*P* < 0.0001). In terms of CA3, the density of PV^+^ neurons did not differ significantly between NNCs and MS-M (*P* > 0.99) but was decreased by ~ 3.5-fold between NNCs and MS-DM (*P* < 0.0001, Supplementary Fig. 2, online resource).

### C1q immunoreactivity correlates with synaptic changes in the CA2 subfield of the MS hippocampus

To determine whether there is a link between the extent of C1q immunoreactivity and synaptic changes in the CA2 of the MS hippocampus, we next performed correlation analyses between the percentage of C1q^+^ area and either the percentage of vGLUT1^+^ or vGAT^+^ or PSD95^+^ or gephyrin^+^ area determined in the CA2 of the same MS hippocampi. Combining the control, myelinated and demyelinated MS hippocampi, we found a significant and positive correlation between the extent of C1q immunoreactivity and the extent of vGLUT1^+^ immunoreactivity (Spearman correlation coefficient, *r* = 0.66, *P* = 0.0001, *n* = 28) but not with the extent of PSD95 immunoreactivity (*r* = 0.28,* P* = 0.15, *n* = 28). In contrast, the percentage of C1q^+^ area was negatively correlated with both vGAT^+^ area (*r* = – 0.55, *P* = 0.002, *n* = 29) and the gephyrin^+^ area (*r* = –0.60, *P* < 0.029, *n* = 29), indicating an association between C1q, gain of excitatory synaptic elements and loss of inhibitory synaptic elements in the CA2 subfield of the MS hippocampus (Fig. 2e). Taken together, these data demonstrate an association between C1q and synaptic reorganization in the CA2 hippocampal subfield of progressive MS donors.

### Enrichment of C1q in the dorsal CA2 subfield in the demyelinated hippocampus of cuprizone-fed mice

**Fig. 3:**
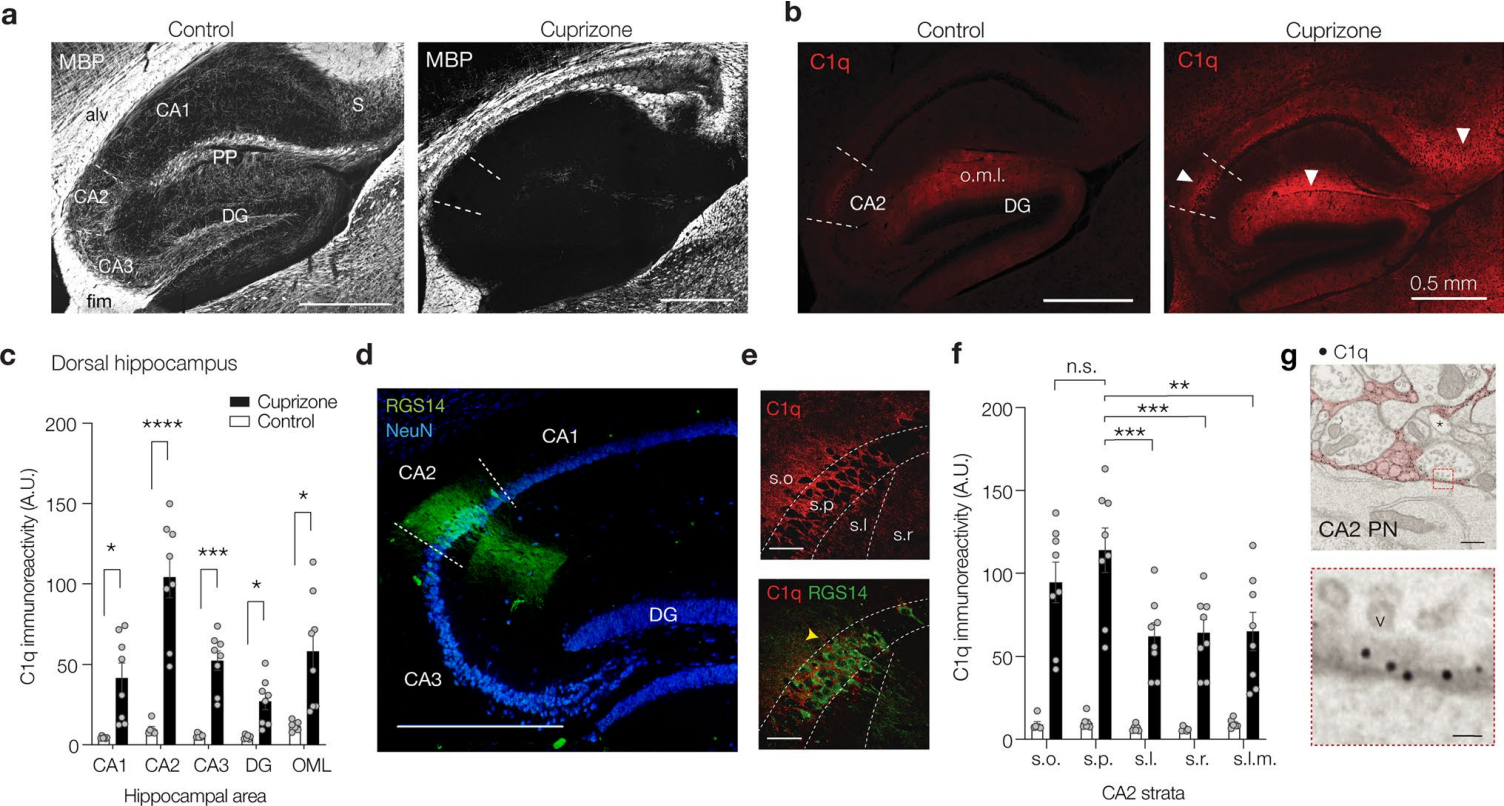
Cuprizone-induced demyelination causes subregion dependent C1q increase. **a** *Left*, Example fluorescence image of a sagittal section of the control dorsal hippocampus for myelin basic protein (MBP-Ab, white) and, *right*, following 9 weeks 0.2% cuprizone treatment. In control, high-intensity signals are present in the white-matter tracts including the alveus (alv) and fimbria (fim) and the myelinated perforant path (PP) fibers. Lower intensity signals are visible in the CA3 and CA2 stratum pyramidale and oriens layers as well as in the dentate granule (DG) molecular layers and subiculum (S). Note the complete loss of all intrahippocampal MBP expression with cuprizone treatment. Scale bar, 0.5 mm. **b** *Left*, control expression of complement factor 1q (C1q-Ab, red). Low levels of C1q are detected in CA2 and DG outer molecular layer (o.m.l.). *Right*, demyelination increases C1q (red) in most hippocampal subfields and with high intensities in CA2, the DG molecular layers and subiculum (white arrows). **c** Population analysis of C1q fluorescence signals in the dorsal hippocampus shows increases in a subregion-specific manner following cuprizone treatment (two-way RM ANOVA, Treatment *F*_1, 12_ = 23.71, *P* = 0.0004; Subregion *F*_2180, 26.16_ = 30.51; *P* < 0.0001 and Subregion × Treatment *F*_4, 48_ = 23.02, *P* < 0.0001, *n* = 6 sections from 3 animals/group). The CA2 region shows the highest immunoreactivity change in comparison to CA1, CA3, the outer molecular layer and the DG (Šidák’s multiple comparisons test, CA1 *P = 0.0261, CA2 ***P = 0.0007, CA3 ***P = 0.0007, DG *P = 0.0157, DG *P = 0.0357). Post-hoc test for regions revealed C1q within CA2 was higher in comparison to all other subregions (Šidák’s multiple comparisons test ***P < 0.0001, data not shown). **d** *Left*, anti-NeuN (blue) in combination with a CA2-specific marker, the anti-regulator of G protein signaling 14 (anti-RGS14, green), staining somata and dendrites of CA2 pyramidal neurons. Scale bar, 500 µm. **e** *Right*, higher-magnification images reveal C1q perisomatic of CA2 neurons (RGS14^+^, green). One RGS14^–^ neuron with perisomatic C1q indicated with an asterisk. Stratum oriens (s.o.), stratum pyramidale (s.p.), stratum lucidum (s.l.), stratum radiatium (s.r.) and stratum lacunosum-moleculare (s.l.m.). Scale bars, 50 µm. **f** Population analysis of C1q intensity across the distinct strata within CA2 reveals a strata-specific cuprizone-induced C1q increase (2-way ANOVA Treatment *F*_1, 60_ = 156.8, *P* < 0.0001, Strata *F*_4, 60_ = 3.69 *P* = 0.0094 and Treatment × strata *F*_4, 60_ = 2.95, *P* = 0.0281, *n* = 8 sections from 4 mice) with after cuprizone treatment s.p. showing higher C1q intensity compared to other strata (Šidák’s multiple comparison tests, versus s.l.m.**P = 0.0012, s.r. ***P = 0.0009, ***s.l. *P* = 0.0005). However, C1q intensities in s.p. and s.o. were similar, *P* = 0.668). Error bars indicate mean ± SEM and grey dots individual section. **g** *Top*, Transmission immunogold EM images of the perisomatic region of a CA2 pyramidal neuron (CA2 PN) of a cuprizone-treated mouse. The anti-C1q immunogold (~ 10 nm black particles) are predominantly within extracellular spaces (false-colored red) near both excitatory synapses (spine head and asymmetric postsynaptic density) and putative inhibitory synapse (red box). Scale bar, 400 nm. Asterisk indicates electron translucent area. *Bottom*, higher magnification of the boxed area showing a putative inhibitory synapse with irregular synaptic vesicles (v) at the CA2 pyramidal neuron soma membrane with C1q-IR gold particles in between membranes. Scale bar, 50 nm

To understand the role of myelin loss and determine the functional consequences of C1q-mediated synapse loss in the CA2 subfield we next investigated the hippocampus in the cuprizone mouse model [36, 66]. Sagittal slices were cut along the dorsal-to-ventral axis of the hippocampus of adult (4-months old) male mice and stained for myelin basic protein (MBP) and compared with age-matched mice treated with cuprizone (0.2% for 9 weeks, Fig. 3a). In the control hippocampus, MBP was densely distributed in the white-matter tracts (fimbria and alveus) and the perforant path. In addition, MBP was also observed throughout the intrahippocampal grey matter regions including CA3 and CA2 (Fig. 3a). Consistent with previous studies with cuprizone [1, 15, 53, 77], myelin was strongly reduced in the white matter regions including the alveus and fimbria and near completely lost in the intrahippocampal grey matter areas (Fig. 3a). This pattern of intrahippocampal myelin loss was highly reproducible across mice and observed along the entire dorsal-to-ventral hippocampal axis (Supplementary Fig. 3, online resource).

Immunofluorescence staining for C1q was performed in the same sections that were stained for MBP. Consistent with previous reports [47, 82], in the control hippocampus C1q (quantified as % immunoreactivity) was found to be detected specifically within the CA2 subfield and the molecular layer of the dentate gyrus (DG) (CA2 and DG o.m.l., Fig. 3b). Following cuprizone feeding, however, C1q immunoreactivity increased widely across the intrahippocampal grey matter and parahippocampal white-matter regions (on average 7.8-fold, *P* < 0.0001, Fig. 3b, c). Quantitative immunofluorescence analysis across the different hippocampal subfields showed that the average C1q^+^ area was significantly higher in CA2 compared to CA1, CA3 and the DG (Fig. 3c). Interestingly, the cuprizone-induced upregulation of C1q followed a gradient along the longitudinal axis with the highest expression level in the dorsal region, lower in the intermediate region and undetectably low in the ventral hippocampus (Supplementary Fig. 3a–d, online resource). To further investigate the prominent CA2 localization of C1q in the demyelinated hippocampus, we stained hippocampal slices from control and cuprizone-fed mice with RGS14, a specific molecular marker for CA2 pyramidal neurons [16]. Co-staining for RGS14 and C1q showed that C1q was predominantly clustered in the stratum pyramidale and oriens of RGS14^+^ neurons at significantly higher levels when compared to the stratum lucidum, radiatum, and lacunosum moleculare (Fig. 3d–f). Interestingly, a few RGS14^–^ neurons in CA2, presumably interneurons, also showed perisomatic C1q (Fig. 3e). Finally, immunogold electron microscopy (EM) for C1q protein confirmed that C1q was mostly detected within the extracellular spaces and matrix, often in close proximity of presynaptic terminals (Fig. 3g).

Thus, in accordance with the demyelinated MS hippocampus (Fig. 1b–d) C1q localization is prominently detected in CA2, and cuprizone-induced demyelination causes a strong complement protein enrichment in the CA2 pyramidal layers.

In the MS hippocampus (Fig. 1 and [51]), models of neurodegeneration [33] and EAE models of demyelination[90] some C1q expression is already detectable before overt signs of pathology or myelin loss. We thus next asked whether the increase in C1q deposition at CA2 precedes or follows the loss of myelin in this region. We quantified the extent of C1q immunoreactivity in relation to MBP immunoreactivity in the CA2 subfield throughout the course of cuprizone feeding (up to 6 weeks). The C1q immunoreactivity increased about twofold from baseline levels at 2 weeks of cuprizone feeding and reached its maximum around 4 weeks. These changes in C1q were mirrored by a loss of MBP immunoreactivity in the CA2 subfield with levels rapidly decreasing by twofold at 2 weeks of cuprizone feeding and reaching maximum loss around 6 weeks (Supplementary Fig. 4a, b, online resource). Correlation analysis showed a significant negative correlation for the CA2 stratum pyramidale layer (*r* = –0.66, *P* < 0.0001, Supplementary Fig. 4c, online resource) supporting a link between the loss of myelin and the C1q increase in CA2 in this model.

Since a classical role of C1q is to tag antigen/(auto)antibody complexes for elimination [68], and anti-myelin antibodies are detected in the serum of models of (auto)antibody-mediated demyelination [20], we next examined whether cuprizone-induced upregulation of C1q in CA2 was associated with serum titers of anti-myelin antibodies at the time when we detect myelin loss in the hippocampus. To test this, we measured anti-myelin oligodendrocyte glycoprotein (MOG) antibody levels in serum from these mice throughout the 6 weeks of cuprizone feeding as well as C57BL/6 mice immunized with human recombinant MOG (hMOG) protein as a technical positive control because hMOG-immunized C57BL/6 mice generate anti-MOG IgG antibodies that are required for the manifestation of clinical disease [20]. As expected, anti-MOG IgG titers were detected in hMOG-immunized C57BL/6 mice. In addition, anti-MOG IgG titers were very low or not detected in control mice (as expected) or cuprizone-fed mice throughout the 6 weeks of cuprizone feeding, including time points when myelin loss is evident (Supplementary Fig. 4d, online resource). These findings indicate that anti-MOG antibodies are not required for the observed demyelination and are unlikely to be the trigger of C1q upregulation in tissue. In summary we show that in the mouse hippocampus, cuprizone feeding triggers a circuit- and cell-specific increase in C1q immunoreactivity that is strongly linked with demyelination but is independent of anti-MOG antibodies, suggesting an antibody-independent role of C1q in the CA2 hippocampal subfield.

### Differential impact of demyelination on excitatory and inhibitory markers

**Fig. 4:**
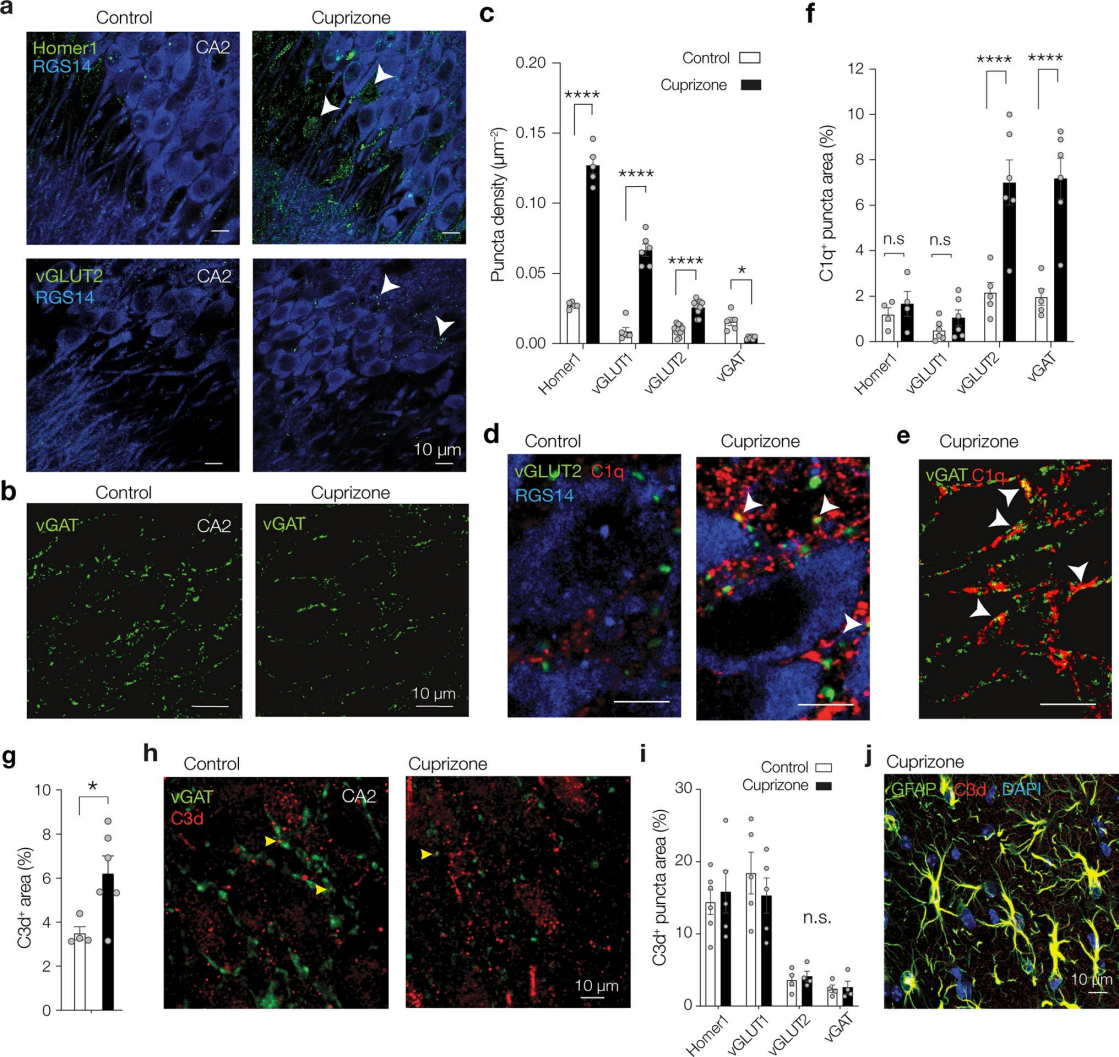
Differential changes in synaptic markers and C1q^+^ co-localization, and C3d^+^ astrocytes in CA2. **a** Example of immunofluorescent staining for RGS14 (blue) and postsynaptic glutamate receptor Homer1 (top, green), the presynaptic excitatory vesicular glutamate transporter 1 (vGLUT1, middle, green) and vGLUT2 (bottom, green) in control (left) and cuprizone hippocampus (right). Note the increase in vGLUT1^+^ puncta in the strata lucidum and radiatum and localization of vGLUT2^+^ puncta in pyramidale and oriens layers. White arrows indicate example puncta included in counting. Scale bar, 10 µm. **b** Example of immunofluorescent staining for vesicular GABA transporter (vGAT, green) overlaid with DAPI (cyan) in the CA2 region of control (left) and cuprizone hippocampus (right). Note the loss in vGAT^+^ puncta in cuprizone hippocampus compared to control hippocampus. Scale bar, 10 µm. **c** Population data for synaptic marker densities reveals a gain in excitatory, but loss of inhibitory synapse markers (Two-way ANOVA Treatment effect *F*_1, 45_ = 427.3, *P* < 0.0001, Treatment × Synapse marker interaction *F*_2, 45_ = 139.4, *P* < 0.0001, followed by Šidák’s multiple comparison test for Homer1 (*t* = 22.95, *df* = 45, ****P < 0.0001), vGLUT1 (*t* = 14.56, *df* = 45, ****P < 0.0001), vGLUT2 (*t* = 5.25, *df* = 45, ****P < 0.0001) and vGAT (*t* = 2.80, *df* = 45, *P = 0.0297). Each group represents *n* = 5–10 sections from 6 control and 4 cuprizone-treated mice. **d** Example triple immunostaining images for RGS14 (blue), C1q (red) and vGLUT2 (green) in control and cuprizone hippocampus. White arrows indicate co-localization of C1q and vGLUT2 (yellow, white arrows). Scale bar, 10 µm. **e** Double immunostaining for vGAT (green) and C1q (red). Note the perisomatic localization of vGAT and co-localization with C1q (yellow color, white arrows). Scale bar, 10 µm. **f** Population data for C1q co-localization (% area overlap) in control (open bars) and cuprizone-treated mice (closed bars). Cuprizone-induced increase in C1q^+^ puncta area significantly co-localizes to vGLUT2 and vGAT signals (Two-way ANOVA treatment × synapse *F*_3, 34_ = 8.64, *P* = 0.0002; Šidák’s multiple comparison test *t* = 5.57, *df* = 34, vGLUT2, ****P < 0.0001, *n* = 5 control and 6 cuprizone, and vGAT *t* = 6.00, *df* = 34,****P < 0.0001, *n* = 5 control and 6 cuprizone) but not Homer1 nor vGLUT1 signals (Šidák’s multiple comparison tests, Homer1; *t* = 0.46, *df* = 34* P* = 0.985, *n* = 4 both groups and vGLUT1; *t* = 0.66, *df* = 34, *P* = 0.945, *n* = 6 both groups). Data represented as mean + SEM with individual sections indicated with circles. **g** Population analysis for the area covered by the C3d^+^ signal in the CA2 subfield reveals a modest increase in cuprizone mice compared to controls (unpaired *t*-test *P = 0.03, *t* = 2.61, *df* = 8, *n* = 4 control and *n* = 6 cuprizone sections from 2 animals per group). **h** Double immunofluorescence staining for C3d (red) and vGAT (green) in the CA2 region of control (left) and cuprizone hippocampus (right). Note the rare localization of C3d with vGAT^+^ puncta (yellow arrows) in the control and cuprizone hippocampus. Scale bar, 10 µm. **i** Population analysis for area of co-localization. Note that C3d significantly clusters with vGLUT1 synapses (two-way ANOVA synapse type *F*_3,29_ = 25.50, *P* < 0.0001, Tukey’s multiple comparison tests vGLUT1 vs. vGLUT2 and vGAT, for both *P* < 0.0001, vGAT versus vGLUT2, *P* = 0.96). In contrast, complement complex C1q is more co-localized with vGLUT2^+^ and vGAT^+^ puncta (c.f. **f**). Overall, cuprizone-induced demyelination did not affect co-localization (two-way ANOVA treatment *F*_1,29_ = 0.028, *P* = 0.87) and neither showed interaction of treatment and synapse types (type × treatment *F*_3,29_ = 0.51, *P* = 0.67). **j** Example of immunofluorescent staining for C3d (red), GFAP (green) and DAPI (blue) in the CA2 region of a cuprizone hippocampus, showing localization of the C3d signal on GFAP^+^ astrocytes. Scale bar, 10 µm

While in the human post-mortem hippocampus we detected C1q both in the soma of neurons and in the perisomatic area of CA2 (the latter most obviously visualized by immunofluorescence staining of the CA2 stratum pyramidale, Fig. 1c), in cuprizone-induced demyelination C1q immunoreactivity in CA2 was distinctly perisomatic particularly in stratum pyramidale and oriens (Fig. 3e, f). To determine the specific changes in synaptic proteins in the demyelinated CA2 we used double immunofluorescence staining for RGS14 (to identify CA2) in combination with markers that identify either glutamatergic synaptic components including the postsynaptic protein Homer1 and the presynaptic vGLUT1 and vGLUT2 (Fig. 4a) or vGAT (Fig. 4b). Quantification of the immunostaining within CA2 revealed a significant increase in the density of excitatory (vGLUT1^+^, vGLUT2^+^ and Homer1^+^) puncta and a concomitant significant reduction in the density of inhibitory (vGAT^+^) puncta (Fig. 4a–c). Notably, unlike the vGLUT1 puncta, which were widely distributed in strata radiatum and oriens, the vGAT^+^ puncta were clustered in the strata oriens and pyramidale, where the highest immunoreactivity for C1q was present and vGAT^+^ puncta apparently contacted CA2 pyramidal neuron cell bodies (Fig. 4b). To examine whether C1q localizes at synapses in the CA2 region during cuprizone-induced demyelination we performed triple labelling for C1q, RGS14 and synapse markers. In comparison to control, cuprizone treatment significantly increased C1q^+^/vGLUT2^+^ and C1q^+^ /vGAT^+^ puncta, but not C1q^+^/vGLUT1^+^ puncta (Fig. 4d-f). Population analysis showed that there was a significant > threefold increased probability that vGLUT2 and vGAT puncta were in contact with C1q and arranged similarly around the CA2 soma (Fig. 4d–f).

Since activation of the classical complement pathway, initiated by the binding of C1q to its target, results in activation of the downstream complement component C3, and since C3 has been involved in elimination of synapses during development [83] and in the MS visual thalamus [90], we next investigated whether also C3 activation products, like C1q, are deposited at synaptic elements in the cuprizone hippocampus. Overall, the percentage of immunoreactivity for the membrane-bound product of C3 activation, C3d, in CA2 was increased by 1.8-fold in cuprizone mice compared to controls (Fig. 4g). The C3d staining showed little co-localization with vGAT^+^ puncta (Fig. 4h) or other synaptic markers (Supplementary Fig. 5, online resources) and the extent of colocalization it did not vary between cuprizone and control mice (Fig. 4i) but it showed extensive co-localization with GFAP^+^ astrocytes (Fig. 4j), likely reflecting the neurotoxic A1 type of astrocytes previously described in MS tissue [45]. Together, these data suggest that cuprizone feeding may cause a C1q-mediated reorganization of synapses in the CA2 pyramidale and oriens layers.

### C1q-tagged synaptic elements localize within microglia/macrophages in the CA2 hippocampal subfield during cuprizone-induced demyelination

**Fig. 5:**
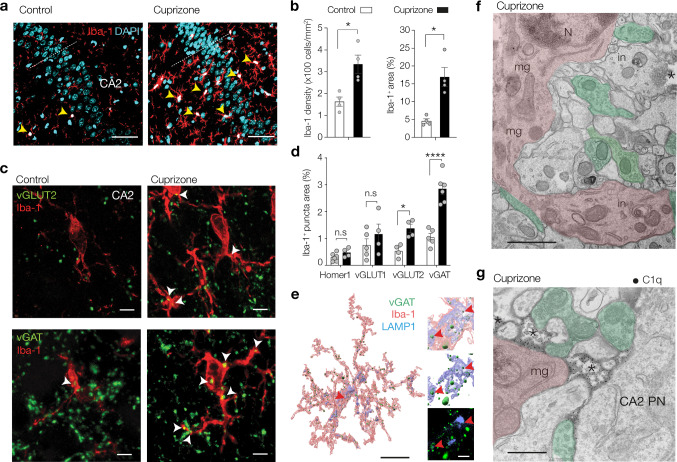
Activated microglia preferentially target vGLUT2^+^ and vGAT^+^ synapses in CA2. **a** Cuprizone increases microgliosis (anti-Iba-1, red) in the hippocampal CA2 subfield, identified with nuclear DAPI stain (blue). Note the increased number and size of microglia/macrophages (yellow arrows). Scale bar, 50 µm. **b** Population analysis of the percentage of DAPI and Iba-1 positive cells (DAPI^+^ Iba-1^+^, white) within area CA2 shows cuprizone doubles the number of Iba-1^+^ cells (two-tailed Mann–Whitney test *P* = 0.0286) and increases the surface area by ~ threefold (two-tailed Mann–Whitney test *P* = 0.0286, *n* = 4 sections from 4 animals). **c** Double immunostaining for Iba-1 and vGLUT2. Note the increased overlap between Iba-1 and vGLUT2 and vGAT (white arrows) in the face of a loss of vGAT puncta. Scale bars, 5 µm. **d** Population data for the overlap of area between Iba-1 and synaptic markers Homer1, vGLUT1, vGLUT2 and vGAT shows a significant cuprizone treatment-induced preference of microglia contact with vGLUT2 and vGAT (Two-way ANOVA Treatment × Synapse marker *F*_3, 30_ = 7.81 *P* = 0.0005, Treatment *F*_1, 30_ = 34.17, *P* < 0.0001, followed by Šidák’s multiple comparison tests, Homer1 *t* = 0.633, *df* = 30, *P* = 0.952; vGLUT1 *t* = 1.42, *df* = 30, *P* < 0.512; vGLUT2 *t* = 2.81, *df* = 30, *P = 0.0337; vGAT *t* = 7.18, *df* = 30, ****P < 0.0001, for all *n* = 4–6 sections from *n* = 4 animals/group). **e** *Left*, Surface rendered image of a Iba-1^+^ and LAMP1^+^ (blue) microglia engulfing vGAT^+^ puncta (red arrow). Scale bar, 10 µm. *Right*, higher magnification of the same cell showing vGAT^+^ puncta (green) inside LAMP1^+^ lysosomes. Bottom raw immunofluorescence images of LAMP1 and vGAT from the region shown above. Scale bar, 1 µm. **f** EM of an activated microglia (*mg*) in the CA2 region. Microglia nucleus (*N*) identified by clumped chromatin. A large process extends towards presynaptic terminals (false green colored). Microglia processes with lysosomes, golgi apparatus, mitochondria and ER contained inclusions (*in*) with phagocytosed debris. Scale bar, 1 µm. **g** Higher magnification of C1q-immunogold EM showing a microglia process with darker cytoplasm (*mg*) in the vicinity of a CA2 pyramidal neuron (PN). C1q containing regions (black asterisks) are near presynaptic terminals (false green colored). Scale bar, 500 nm

Complement-tagged synapses are eliminated via phagocytoses by microglia during development, adulthood, normal ageing, neurodegeneration, and demyelination of the visual thalamus [33, 82, 83, 89, 90]. To test whether microglia/macrophages engulf C1q-tagged synaptic elements in CA2, we first quantified changes in the number of cells positive for ionized calcium-binding adaptor molecule 1 (Iba-1), a marker for microglia/macrophages. Quantification of Iba-1^+^ cells in CA2 showed a significant twofold increase in number as well as a significant threefold increase in the area covered by the Iba-1^+^ cells in the cuprizone-treated mice compared to controls (Fig. 5a, b). Double immunofluorescence labeling of Iba-1 with either Homer 1, vGLUT1, vGLUT2 or vGAT showed a basal level of co-localization of Iba-1 with each of the synaptic element markers in the control hippocampus, likely reflecting ongoing surveillance of microglia. However, cuprizone feeding induced a specific increase in Iba-1^+^/vGLUT2^+^ and Iba-1^+^/vGAT^+^ puncta (Fig. 5c, d). 3D rendering also showed microglial/macrophage processes surrounding vGAT^+^ puncta within LAMP1^+^ lysosomes (see example in Fig. 5e), pointing to the engulfment of synaptic elements by myeloid cells in the CA2 area. In line, immunogold-EM for C1q protein in cuprizone-fed mice showed large microglial processes engulfing electrodense element in proximity to presynaptic terminals (Fig. 5f) or touching C1q-labeled synapses in the CA2 hippocampal subfield (Fig. 5g), further supporting the close association and engulfment of synaptic elements by myeloid cells in the CA2 area.

### Cuprizone selectively reduces feedforward inhibition of CA2 pyramidal neurons

**Fig. 6:**
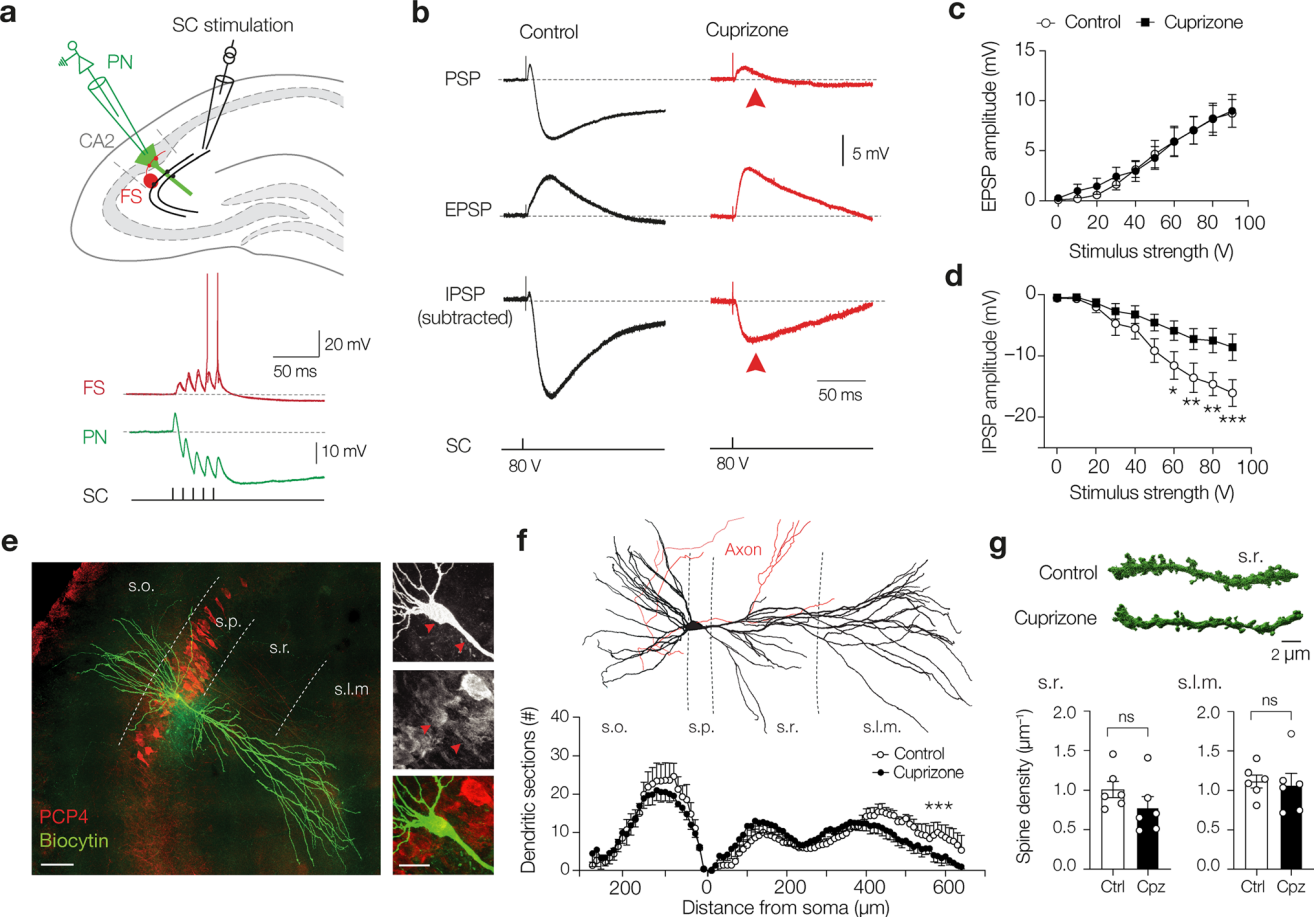
Cuprizone impairs CA3 to CA2 feedforward inhibition. **a** Top, schematic of the recording configuration of a CA2 pyramidal neuron (PN, green) overlaid with a schematic of a stimulation pipette for SC axon fibers (SC). Bottom, whole-cell recordings of a fast-spiking (FS) interneuron showing SC-evoked temporal summation with a gradual increase in AP generation. In contrast, SC-evoked CA2 PNs (green) are shunted by the strong feedforward inhibition. SC axons were activated with 5 × 60 V pulses (100 Hz, 0.3 ms duration). **b** Top, SC-evoked postsynaptic potentials in CA2 PNs from control (grey traces) and cuprizone (red traces). Middle, same recordings after bath application of the GABA_A_ and GABA_B_ antagonists (CGP 35,348 and SR 95,531, respectively) uncovering excitatory postsynaptic potentials (EPSP, light gray). Bottom, digitally subtracted traces (control–EPSP) revealing the underlying IPSP. Note the reduced amplitude of the IPSP in CA2 PNs in slices from cuprizone-treated mice (red arrows). **c** Population data for isolated EPSPs and IPSP as a function of stimulus strength (0–90 V). The EPSPs amplitudes were unaffected by cuprizone treatment (mixed-effects model RM ANOVA, Treatment *F*_1, 11_ = 0.062 *P* = 0.809, *n* = 5 control and 8 cuprizone neurons from 6 mice/group). **d** IPSP peak amplitudes were significantly reduced (mixed-effects model RM ANOVA Treatment *F*_1, 13_ = 8.71, *P* = 0.0112, Treatment × stimulus interaction *F*_9, 96_ = 4.95, *P* < 0.0001, *n* = 8 neurons from 6 mice/group) and increased at stimulus intensities > 60 V (Šidák’s multiple comparison test 50 V *P* = 0.071, 60 V *P = 0.0146, 70 V **P < 0.0053, 80 V **P = 0.0017 and 90 V ***P = 0.0010). **e** Immunofluorescence image of a biocytin-filled PN (streptavidin-biocytin, green), which was positive for the CA2 marker PCP4 (red). Scale bar, 100 µm. Inset scale, 25 µm. Red arrows indicate somatic and dendritic PCP4 expression. **f** Top, example 3D reconstruction of a CA2 pyramidal neuron from a control cell. Soma and dendritic branches in black, axon in red. Bottom, Scholl plot analysis (*n* = 5 control neurons, *n* = 6 cuprizone), revealing a differential distribution of dendrites in the apical dendrites (mixed-effect RM ANOVA Treatment × Scholl ring interaction *F*_64, 530_ = 1.85, ***P < 0.0001, Treatment *F*_1, 9_ = 0.088, *P* < 0.77) but not in basal dendrites (Treatment × Scholl interaction *F*_27, 222_ = 0.70, *P* = 0.863). **g** 3D surface-rendered example images of CA2 s.r. dendritic branches. Spine quantification revealed similar densities in branches from s.r. and s.l.m. (Mann–Whitney tests *P* = 0.0931 and 0.484, respectively, *n* = 6 mice/group)

Our immunofluorescence analyses were performed with synaptic markers but do not indicate whether synapses are functionally increased or decreased. What are the consequences of C1q-tagged and microglia/macrophages phagocytosed for information processing in the CA2 circuit? CA2 PNs receive a strong excitatory drive from layer 2 medial and lateral entorhinal cortex pyramidal neurons at their distal dendrites in the lacunosum moleculare and in the stratum pyramidale and oriens layers but weak excitation from both CA3 Schaffer collateral (SC) and DG mossy-fibers at the proximal dendrites [3, 8, 38, 52]. In addition, in the s.p. and s.o. regions, where C1q immunoreactivity was markedly increased (Figs. 4 and 5), vGLUT2 reflects glutamatergic innervation from the medial septum diagonal band complex and the hypothalamic supramammillary nucleus [26, 29], whereas the vGAT is, in part, associated with excitation of fast-spiking parvalbumin (PV)^+^ interneurons conveying a strong feedforward inhibition from the CA3 SC axons to CA2 [3, 44, 52, 61] (Fig. 6a). CA2 PV^+^ interneurons are furthermore subjected to neuromodulation from the hypothalamic paraventricular nucleus (PVN) and supraoptic nucleus (SON), playing a critical role in encoding social learning by affecting the plasticity of PV interneurons [44, 61, 62]. To test whether the gain of vGLUT or loss of vGAT puncta causes functional changes in the CA2 subfield we electrically stimulated SC axons while recording from CA2 PN somata in transverse slices from the dorsal hippocampal region from control and cuprizone-fed mice (6 weeks for 0.2% cuprizone, Fig. 6a). All recorded neurons were simultaneously filled with biocytin and post-hoc stained with RGS14 or PCP4. About 80% of the recorded neurons (*n* = 29/36) were unequivocally CA2 PNs and included for further analysis for their properties.

In CA2 PNs in control slices, SC stimulation produced a brief depolarization followed by a strong inhibitory potential (Fig. 6a [8]). Functional changes in inhibitory and excitatory inputs were quantitatively examined using a single brief SC stimulus with varying levels of voltage strengths (0–90 V). The depolarizing peak of the postsynaptic potential (PSP) did not differ between the two groups across the range of stimuli (mixed-effect ANOVA Treatment *F*_9, 171_ = 0.688, *P* = 0.548, at 90 V; control PSP amplitude, 3.20 ± 0.59 mV, *n* = 12, vs. cuprizone PSP 4.04 ± 0.64, *n* = 9, Fig. 6b, data not shown). To distinguish between the monosynaptic glutamate receptor and disynaptic GABAergic receptor activation from the SC pathway we applied CGP 35,348 (antagonist of GABA_B_ receptors, 20 µM) and gabazine (SR 95,531, a selective GABA_A_ antagonist, 3 µM). Analysis of the pharmacologically isolated EPSP revealed that SC activation in cuprizone-treated mice produced similar peak amplitudes across the entire range of stimuli (mixed-effect RM ANOVA, Treatment *P* = 0.809, Fig. 6b, c). In striking contrast, the IPSP peak amplitudes (subtracting the control PSP with the EPSP), were significantly reduced (Fig. 6b, d). In addition, calculating the relative contribution *within* recordings (EPSP/IPSP amplitude ratios) confirmed a significant loss of net inhibition of CA2 PNs from cuprizone-treated mice (control ratio, 0.64 ± 0.06 versus cuprizone ratio 1.11 ± 0.15, two-tailed Mann–Whitney test *U* = 7, *P* = 0.0064, *n* = 8 neurons from 6 mice/group). About ~ 80% of the GABAergic cells in the CA2 stratum pyramidale are parvalbumin-positive (PV^+^) interneurons [4]. To test whether the reduced feedforward inhibition is associated with interneuron cell loss we counted the number of PV^+^ interneurons in the CA2 region using double immunofluorescence staining with PV and RGS14. The PV^+^ interneuron density in the demyelinated hippocampus was not different following 7 weeks cuprizone treatment (Supplementary Fig. 6, online resource). To test whether cuprizone induces morphological changes of CA2 PNs we analyzed the post-hoc reconstructed RGS14^+^ or PCP4^+^ neurons from biocytin-filled neurons (Fig. 6e). The total RGS14^+^ area (within s.p. and s.o.) was not different following cuprizone treatment (Supplementary Fig. 6, online resource). Interestingly, while detailed reconstructions of biocytin-filled CA2 pyramidal neurons showed that the total dendritic length was not different (control, 4.28 ± 0.75 mm, *n* = 5 vs. cuprizone 3.28 ± 0.58 mm, *n* = 6, Mann–Whitney test U = 9, *P* = 0.329), Scholl analysis showed that cuprizone treatment was associated with a lower number of sections in the s.l.m region (*P* < 0.0001, Fig. 6f). Using high-quality filled CA2 PNs we further examined the spine density along branches in s.r. and s.l.m. Consistent with the conserved EPSP amplitudes, these data showed no change in spine density (Fig. 6g).

Together, the functional recordings show that local CA3 to CA2 feedforward inhibition is strongly impaired, and consistent with the C1q-tagged and microglia/macrophage stripped vGAT^+^ release sites (Figs. 4, 5).

### Demyelination induces reduced excitability of CA2 pyramidal neurons

**Fig. 7:**
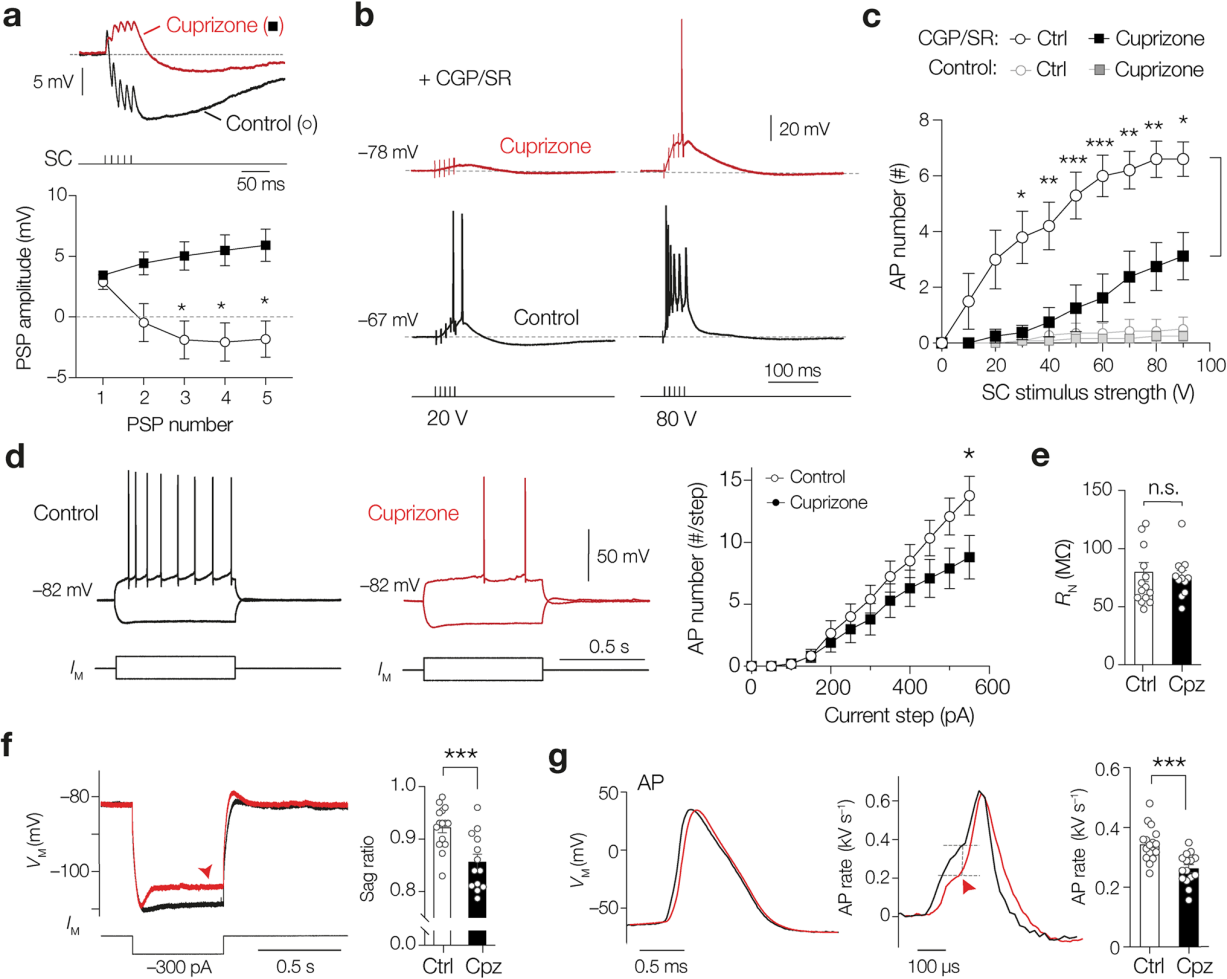
Cuprizone-induced demyelination reduces synaptically evoked- and intrinsic action potential output. **a** Top, example traces of SC-evoked postsynaptic potentials, reflecting small excitation followed by feedforward inhibition (control) at 100 Hz train of subthreshold 60 V stimuli. Note the strong accumulation of inhibitory potentials in control CA2 neurons but not in cuprizone neuron. Bottom, population data of the average peak amplitude responses (positive deflection relative to resting potential) as a function of stimulus pulse number (2-way RM ANOVA stimulus *F*_1244, 25_ = 2.53, *P* = 0.12, Treatment *F*_20, 80_ = 16.26, *P* = 0.0076, Treatment × stimulus interaction *F*_4, 80_ = 8.477, *P* < 0.0001) with significantly increased amplitudes for the 3^rd^ to 5^th^ stimulus (For all, Šidák’s multiple comparisons test *P* < 0.05, *n* = 13 control and 9 cuprizone neurons from 6 mice/group). **b** Left, example traces for SC evoked excitation at 100 Hz in the presence of CGP 35,348 and SR 95,531. Note the low spike probability in the recordings from cuprizone treatment (top, red traces). **c** Population data summarizing AP number per train across the range of stimuli in physiological extracellular solution (control, gray lines and symbols) or with blocked inhibition (CGP/SR, black open and closed symbols). Cuprizone suppressed SC-mediated CA2 PN spike output in stimulus strength dependence (two-way ANOVA Treatment *F*_1, 15_ = 14.79, *P* = 0.0016 Treatment × stimulus *F*_9, 135_ = 4.183, *P* < 0.0001). **d** Characteristic CA2 pyramidal neuron spike generation of a control CA2 and cuprizone-treated CA2 PN, showing delayed action potentials and near threshold ramp depolarization. Current-frequency (*I-f*) plots for CA2 PNs shows cuprizone reduces the maximum spike output rate (two-way RM ANOVA Treatment *P* = 0.1799, Treatment × current step interaction *P* = 0.0044, Holm-Šidák’s multiple comparison tests, for 0 to 500 pA *P* > 0.088, 550 pA *P = 0.022, cuprizone, *n* = 10 and *n* = 12 control neurons from 5 mice/group). Data represent mean ± SEM. **e** Input resistance (*R*_N_) was similar between groups (two-tailed *t*-test *P* = 0.898, *n* = 10 cuprizone and 15 control neurons, 5 mice/group). **f** Cuprizone increased the sag ratio to hyperpolarized steps near –110 mV (unpaired t-test, two-tailed *t* test ***P = 0.0007, *n* = 10 cuprizone and 15 control neurons, 5 mice/group). **g** Action potentials are specifically slower at their initial rate d*V*/d*t* (inset), reflecting axonal charging of the somatodendritic domain (two-tailed *t* test ***P = 0.0002, *n* = 15 cuprizone and 17 control neurons, 5 mice/group). For **d**, **e**, **f,** Bars represent mean ± SEM with circles individual cells

The switch from a net feedforward inhibition to excitation was strikingly visible when stimulating SCs with a burst of 5 stimuli (@100 Hz, Fig. 7a). In control CA2 PNs, the PSP peak amplitudes summated highly *sublinear* and the 5^th^ peak potential amplitude was on average ~ 5 mV more hyperpolarized relative to the first peak potential due to slow inhibitory potentials [8] whereas after cuprizone treatment the PSP peak amplitudes summated and increased by ~ 1 mV relative to the first peak (Fig. 7a). The strong feedforward inhibitory drive from CA3 typically limits CA2 spike output and in accordance, APs were only observed in 2/14 control CA2 PNs at the maximum strength of 90 V (on average 0.5 ± 0.43 APs, Fig. 7b, c). A similar low probability for spike output of CA2 PNs was noted in cuprizone treated mice (1/10 recordings, on average 0.22 ± 0.22 APs, mixed-effect ANOVA, Treatment *F*_9,207_ = 0.297, *P* = 0.975, Fig. 7b, c). As expected from GABA_A/B_ receptor block, in control CA2 PNs a larger number of APs were evoked with SC stimulation (> 6 APs from 30 V, Fig. 7b, c). In contrast, however, following cuprizone treatment CA2 PNs showed a ~ threefold lower spike output rate (Fig. 7b, c). Thus, with the lower dynamic range in CA2 feedforward inhibition, the remaining glutamate-mediated synaptic drive from CA3 neurons in the demyelinated hippocampus produces only a weak spike output.

To examine the role of intrinsic excitability in the deficit of spike generation in CA2 PNs, we used direct depolarizing current injections at the soma. While the resting membrane potentials were not different (control, – 81.4 ± 2.0 mV, *n* = 13 versus – 82.6 ± 1.2, *n* = 11, unpaired *t* test *P* > 0.60) and CA2 PNs showed the characteristic delay in spike generation as described previously [8], the maximum firing rate with higher current injections was lower in CA2 PNs from cuprizone-treated mice (Fig. 7d). The decrease in excitability was not associated with a predicted reduction in neuronal input resistance (unpaired *t* test *P* > 0.72, Fig. 7e). The minimum current to evoke AP generation was neither different between groups (Mann–Whitney test *U* = 51, *P* = 0.565, *n* = 10 cuprizone and 12 control neurons, 5 mice/group). Interestingly, the sag ratio, as measured by the degree of depolarization upon hyperpolarization steps to – 110 mV revealed that CA2 PNs from cuprizone-treated animals exhibited significantly increased depolarizing amplitudes upon hyperpolarization, suggesting an increased density of dendritic hyperpolarization-activated cyclic nucleotide-gated (HCN) conductance (Fig. 7f). Finally, a detailed analysis of single APs showed that while most AP properties were similar the first rate-of-rise component, reflecting the local axonal charging of the spike [39], was significantly decreased by on average ~ 100 V s^–1^ in cuprizone-treated mice (Fig. 7g). Taken together, these data suggest that cuprizone-induced demyelination increases the HCN conductance and reduces spike generation at the axonal output site ultimately limiting spike output from CA2 pyramidal neurons.

### Social memory is impaired by cuprizone treatment

**Fig. 8:**
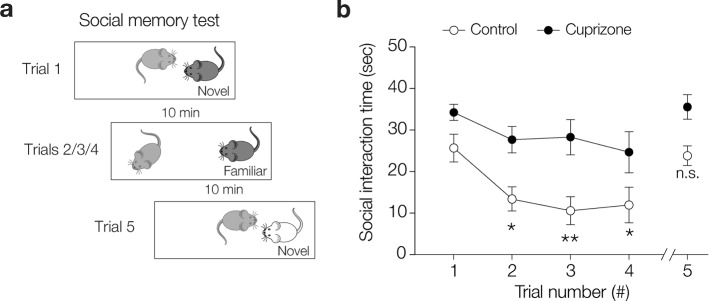
Cuprizone-induced demyelination impairs social memory. **a** Schematic of the five-trial social memory test. Subject animals (light grey) were placed in a cage and after 15 min an unfamiliar novel mouse was introduced for a 1-min trial duration. The stimulus reintroduced 4 times (trials 2 to 4) with each 10 min inter-trial intervals. Subject mice are expected to show habituation based on memorizing social cues. At the 5^th^ trial a novel mouse was introduced to measure dishabituation. **b** Population analysis for the total time spent by the subject mouse socially investigating the stimulus mouse (anogenital sniffing, approaching and close proximity behaviors). Cuprizone treatment caused a significantly different habituation behavior (two-way repeated measures ANOVA, Treatment *F*_1,20_ = 13.72, *P* = 0.0014, Trial × Treatment *F*_4,80 =_ 0.78, *P* = 0.779). Control mice habituated in trials 2, 3 and 4 to the familiar mouse (Bonferroni’s multiple comparisons tests; 1 vs. 2, *t* = 4.39, *P = 0.010; 1 vs. 3, *t* = 4.91, **P = 0.0049; 1 vs. 4, *t* = 3.69, *P = 0.034, respectively) and dishabituated in trial 5 (1 vs. 5, *t* = 0.66, *P* > 0.99, *df* = 10*, n* = 11 mice). In contrast, cuprizone mice did not show habituation to the familiar mouse (Bonferroni’s multiple comparisons tests; 1 vs. 2, *t* = 2.01, *P* = 0.56; 1 vs. 3, *t* = 1.16, *P* > 0.99; 1 vs. 4, *t* = 1.83, *P* = 0.778 and 1 vs. 5, *t* = 0.33, *P* > 0.99, for all *df* = 10 and* n* = 11 mice). Data show mean ± SEM

The dorsal CA2 area receives unique subcortical inputs signaling emotional states and is a circuit for social memory formation [31], mediated by suppressing the PV-mediated feedforward inhibition and gating CA2 output to the ventral CA1 area [31, 44, 49]. Previous studies showed that cuprizone treatment increases social behaviors in a resident-intruder paradigm, but negatively impacts complex motor tasks and hippocampal-dependent spatial learning [15, 30]. To investigate directly whether the CA2-mediated encoding of social memory is affected we used the five-trial social memory test [31]. The results showed that cuprizone significantly affected social memory (two-way RM ANOVA *F*_1,20 =_ 13.7, *P* = 0.0014, *n* = 11 mice/group, Fig. 8). Whereas control mice significantly reduced the time investigating the familiar conspecific, indicating social memory, they dishabituated when confronted with a novel mouse (Bonferroni’s multiple comparison tests Trials 2–4, *P* < 0.034, Fig. 8, Supplementary Movie S1, online resource). In contrast, cuprizone treatment for seven weeks caused a lack of habituation (Bonferroni’s multiple comparison tests for Trials 2–4 versus Trial 1, *P* > 0.60, *n* = 11, Fig. 8, Supplementary Movie S2, online resource), and neither did mice dishabituated with a novel mouse (*P* > 0.999, Trial 5 vs Trial 1, *n* = 11, Fig. 8). The deficit in social memory was not a general learning impairment or reflecting changes in motor activity; automated analysis of the behavior of mice in a discrimination learning task, in which food pellets were provided as a reward [67], showed that control and cuprizone-treated mice spend an equal amount time exploring the cage and both groups were able to learn the task with cuprizone-treated mice acquiring the task even slightly faster (*P* = 0.192 and *P* < 0.0001, respectively, Supplementary Fig. 7, online resource).

Together, the findings indicate that cuprizone-induced demyelination causes deficits in learning in specific domains and, in line with the impaired CA2 inhibitory circuit, social cognition was found to be impaired.

## Discussion

In this study, we identified the CA2 hippocampal subfield as a region for C1q-associated loss of inhibitory synaptic elements both in the MS hippocampus and cuprizone-induced demyelination. Using post-mortem MS tissue collected at rapid autopsy we found that C1q immunoreactivity is most prominently increased in CA2, associated with a loss of vGAT^+^ puncta, and its upregulation correlated with hippocampal atrophy and cognitive deficits. The observed loss of vGAT^+^ signals in CA2 and CA3 in the MS hippocampus may in part reflect interneuron loss, as we show with NeuN staining and more specifically with PV staining, but it remains to be examined whether synaptic protein expression is changed and whether synapse numbers are reduced. Nonetheless, our findings suggest that within the neuronal circuits in the MS hippocampus inhibition is strongly diminished. This is in line with a recent neuropathological examination of the post-mortem cortex from progressive MS patients showing selective loss of PV and somatostatin interneurons together with a reduced number of GABAergic synapses but preserved glutamatergic synapses [92].

Although extremely valuable, the human MS post-mortem hippocampal tissue offers only an endpoint snapshot of a complex pathological cascade. Animal models which reproduce the synaptic alterations seen in MS are a prerequisite to elucidating the mechanisms and functional consequences of hippocampal changes. While acute EAE models reflect the contribution of acute inflammation to pathology [42], dietary cuprizone feeding in mice induces hippocampal demyelination with little inflammatory lesions [36, 66], resembling some of the histopathological presentations of grey matter lesions in MS patients [21]. Using EM, confocal microscopy, and electrophysiology in the hippocampus of the cuprizone model of demyelination, we showed that C1q was selectively enriched at vGLUT2^+^ and vGAT^+^ puncta, which were engulfed by microglial processes resulting in a substantial reduction in the number of only GABAergic synaptic puncta in the stratum pyramidale and oriens of CA2 and loss in GABAergic inhibition. That specifically GABAergic synapses are tagged by C1q, engulfed and eliminated by microglia independently of anti-MOG antibodies in the cuprizone model, disconnects the process of C1q-mediated elimination of synapses from the classical role of C1q as recognition molecule of T-cell mediated antigen/antibody complexes that typically occurs in autoimmune diseases as part of the inflammatory response. This is in line with the current knowledge that MOG-antibody associated demyelinating disease is different from MS and cortical demyelination in MS occurs independent of anti-MOG antibodies [41]. Nonetheless, the association between the amount of C1q immunoreactivity localized in CA2 and the amount of MBP reduction in this region, in mice (Supplementary Fig. 23, online resource) and in MS (Fig. 1d), suggest that C1q-mediated processes are at least concomitant to myelin loss. Whether the increase in C1q contributes to demyelination or whether it is an independent event remains unresolved. There is evidence for both scenarios: on the one hand, microglia are the main producers of C1q [19, 88] and are the main driver of demyelination in cuprizone-fed mice [48], pointing to the possibility that microglia-derived C1q could contribute to microglia-dependent demyelination by tagging myelin and possibly result also in bystander tagging of nearby synapses. On the other hand, the process of microglia-mediated elimination of C1q-tagged synapses is reminiscent of observations during early development [83], adulthood [89], normal ageing [82] and neurodegeneration [33], all of which are conditions that do not involve demyelination, indicating that C1q-mediated elimination of synapses may be an activity-dependent and coordinated event which is independent of demyelination.

Recent work from Werneburg et al. [90] showed that synaptic material is tagged by complement C3 (not by C1q) and is engulfed by microglia in the retinogeniculate system of models of demyelination and in the visual thalamus of MS patients before the onset of clinical disease and before overt signs of demyelination. Although we also observed a higher density of C3d in the CA2 hippocampal subfield of cuprizone mice compared to controls, this complement activation product mainly localized to GFAP^+^ astrocytes. This is consistent with recent work showing that microglia-derived C1q (together with IL-1α and TNF) induce astrocytes to transition to a more reactive phenotype, including induction of astrocytic C3[45]. In contrast to the Werneburg et al. study [90], we found that C1q but not C3 is deposited at discrete types of synapses that are engulfed by microglia in the MS [51] and cuprizone hippocampus, suggesting that different complement pathways may be at play in different neural circuits in the mouse and human brain. Interestingly, in the demyelinated CA2 region and within the stratum pyramidale and oriens layers, C1q specifically targeted and engulfed vGLUT2^+^ and vGAT^+^ puncta but not Homer1^+^ or vGLUT1^+^ puncta. Whilst immunoreactivity for vGLUT1 and Homer1 was strongly increased across intrahippocampal regions (in cuprizone and MS hippocampi) our whole-cell recordings for Schaffer-collateral responses did not show a change in EPSP input strength. The discrepancies between preserved excitatory transmission and spine density but increased glutamatergic synapse markers remain to be further investigated. One possible explanation is that the upregulation of glutamatergic synapse markers are non-neuronal and represent reactive astrocytes. An upregulation of Homer1 also is seen in reactive astrocyte types that switches astroglial signalling pathways during inflammatory conditions [6]. Furthermore, increased vGLUT1 expression may represent vesicle accumulation in injured axons, which develop as spheroids in both cuprizone and MS [72].

An important question that arises from the present findings is what molecular and/or activity-dependent mechanisms determine which synapses are targeted and which are spared by C1q? At the molecular level, a recent study in the retinogeniculate system demonstrated that a “don’t eat me” signal, such as CD47, is required to prevent excess pruning of synapses during development [43]. CD47 could directly inhibit phagocytosis by binding to its receptor, SIRPα, on microglia/macrophages [54, 55] and it has also been shown to prevent engulfment of cells opsonized with “eat me” flags, such as complement, showing that it can override these signals [56]. In addition, C1q was copurified with synaptosomes containing markers of apoptosis [25], suggesting that synaptic pruning may involve some of the same molecular triggers as the complement-mediated enhanced clearance of apoptotic cells that occurs as part of a homeostatic (non-phlogistic) process in the periphery [69]. Whether “don’t eat me” signals on spared synapses or “eat me” (i.e. apoptotic) signals on tagged synapses are involved in the engulfment of complement-tagged inhibitory synaptic elements in the rodent and/or MS demyelinated hippocampus remains to be determined. One additional mechanisms by which RGC synapses are eliminated during development involves neuronal activity, with microglia engulfing less active RGC inputs [74], in line with the knowledge that less active or ‘weaker’ inputs are pruned and lose territory as compared to those inputs that are ‘stronger’ or more active, which elaborate and strengthen [12]. The changes in ascending and descending inputs from the hippocampus as well as the intrahippocampal activity and the role of C1q herein remain to be further examined. For example, using the C1q knockout mice [5] or complement inhibitors [78] in combination with cuprizone treatment will allow resolving its causal and specific contribution in changing the hippocampal circuits.

### Functional reorganization of demyelinated intrahippocampal circuits

In the cuprizone model, the loss of GABAergic terminals was not limited to specific CA subfields but more widespread. Indeed, cuprizone treatment affects besides the CA2-mediated social memory (this study, Fig. 6) also spatial navigation [15], typically mediated by synaptic plasticity in the CA1 and CA3 areas in the dorsal hippocampus in rodents [73]. Although CA2 represents only a small region of the CA pyramidal layer, emerging evidence indicates that CA2 is genetically, molecularly, and physiologically unique and acts as a hub controlling subcortical and intrahippocampal information processing [13], and could represent a novel target for therapeutic treatment [7]. Unlike CA1 and CA3, the CA2 PNs receive strong long-range extrahippocampal and input from both layer 2 medial and lateral entorhinal cortex pyramidal neurons at their distal dendrites [3, 8, 38, 52] and direct vGLUT2-mediated inputs from the medial septum diagonal band complex and the supramammillary nucleus [26, 29]. How the myelin loss from local inhibitory interneurons and/or excitatory long-range projections in combination with the synaptic reorganization impacts the temporal structure of activity in CA2 and the feature of encoding needs to be further determined, e.g. by using in vivo recordings and cell-selective optogenetic approaches. Furthermore, it would also be important to determine how activity-dependent plasticity of inhibitory synapses is affected. Data on the electrophysiological consequences of myelin loss in the hippocampus is scarce and the neuronal changes are likely to be complex. Indeed, our results showed that the strong reduction in feedforward inhibition was associated with a hypo-excitability of the CA2 pyramidal neurons (Fig. 7) which could represent a compensatory change. These findings are in line with recent longitudinal in vivo Ca^2+^ imaging of CA1 PNs over the course of 7 weeks cuprizone/rapamycin treatment reporting a reduced firing rate, rapidly recovering during remyelination [11]. In contrast, using EEG recordings in the hippocampus of awake and freely moving mice Hoffmann et al. [32], reported large-amplitude spikes and seizure activity after 9 weeks of cuprizone treatment. The present finding of an impaired inhibitory circuit in the CA2 subregion (Fig. 4) could provide a cellular basis giving rise to hippocampal seizure activity. Consistent with this conjecture, Boehringer, and colleagues [3] showed that the CA2 region acts as a central hub to balance excitation and inhibition across CA1 and CA3 areas. Using chemogenetic silencing of the CA2 neurons transformed sharp-wave ripple activity into seizure-like discharges [3]. Such widespread synchronization of inhibitory activity may in part be dependent on the strong feedforward inhibition by fast-spiking CA2 interneurons. The CA2 region contains a high density of PV^+^ interneurons [4, 50] with a unique morphology and mid-range axonal projections targeting CA1 and CA3 pyramidal layers. Interestingly, a large fraction of the intrahippocampal myelination represents sheaths that are wrapped around GABAergic interneuron axons, mostly including the PV^+^ axons [50, 81]. Whether PV^+^ interneuron excitability changes with demyelination and what triggers C1q upregulation to prune GABAergic release sites is not well understood but is an important area for further research.

### A role of CA2 circuits in cognitive impairments?

The importance of anatomical parcellation and functional subspecialization of the hippocampal subfields in cognitive problems in MS had already been brought forward by diffusion tensor MRI studies of atrophy and connectivity in MS [59]. For example, CA1 atrophy is a prediction for verbal memory performance [46, 63, 79], while CA2/3 atrophy underlies depressive symptoms [22], and changes in the dentate gyrus enlargement may explain poor cognitive performance [70]. The subcortical areas with which the CA2 pyramidal neurons and interneurons relate to are involved in emotional regulation and include the hypothalamus, amygdala, and septum. To what extent the CA2 connectivity and the local PV interneurons are affected in MS and whether its role in social memory consolidation is homologous to a rodent is unknown and remains to be further examined with high-resolution MRI imaging enabling the parcellation of small CA2 area and by performing further detailed molecular analysis of area CA2 in the postmortem brain. That CA2 in the human hippocampus is critical to cognition, is supported by a meta-analysis of post-mortem studies, revealing that PV^+^ interneuron loss specifically within the CA2 is one the strongest predictors for schizophrenia and mood disorders [37]. Both social cognition and facial emotion recognition, have been identified as domains that are affected in MS patients and associated with reduced social activities and a burden for the quality of life [10, 24]. Social cognition steers the ability to interpret and interact with the mental states of others and is a core psychological skill to maintain relationships and social support. This capacity is crucially important for people with MS to mitigate the disease and recent studies showed that a decline in social cognitive skills negatively impacts the quality of the life for MS patients. Our findings of a disrupted inhibitory CA2 circuit related to impaired memory for conspecifics in the demyelinated hippocampus resemble the changes in CA2 seen when deleting the psychiatric-disease related gene 22q11.2 causing also an impaired CA3 to CA2 feedforward mediated inhibition as well as a reduced CA2 PN spike output resulting in an impaired social memory [62]. Altogether, our work adds to the emerging evidence of subspecialization of the hippocampal subfields in specific cognitive domains which may also help explain subfield-specific susceptibility to injury in MS.

## Supplementary Information

Below is the link to the electronic supplementary material.

Supplementary file 1 (DOCX 6722 kb)

Supplementary file 2 (MP4 4562 kb)

Supplementary file 3 (MP4 4988 kb)
