# Supplementary material for: Complement-associated loss of CA2 inhibitory synapses in the demyelinated hippocampus impairs memory
Source: Acta Neuropathol. 2021 Jun 25;142(4):643–67. doi: 10.1007/s00401-021-02338-8 (PMC8423657; doi:10.1007/s00401-021-02338-8)
Supplement: Supplementary file 1 — (DOCX 6722 kb) [file 401_2021_2338_MOESM1_ESM.docx]

**Supplementary Information**

**Complement-associated loss of CA2 inhibitory synapses in the demyelinated hippocampus impairs memory**

Valeria Ramaglia*, Mohit Dubey, M. Alfonso Malpede, Naomi Petersen, Sharon I. de Vries, Shanzeh M. Ahmed, Dennis S.W. Lee, Geert J. Schenk, Stefan M. Gold, Inge Huitinga, Jennifer L. Gommerman, Jeroen J.G. Geurts and Maarten H.P. Kole*.

**Supplementary figures**

**
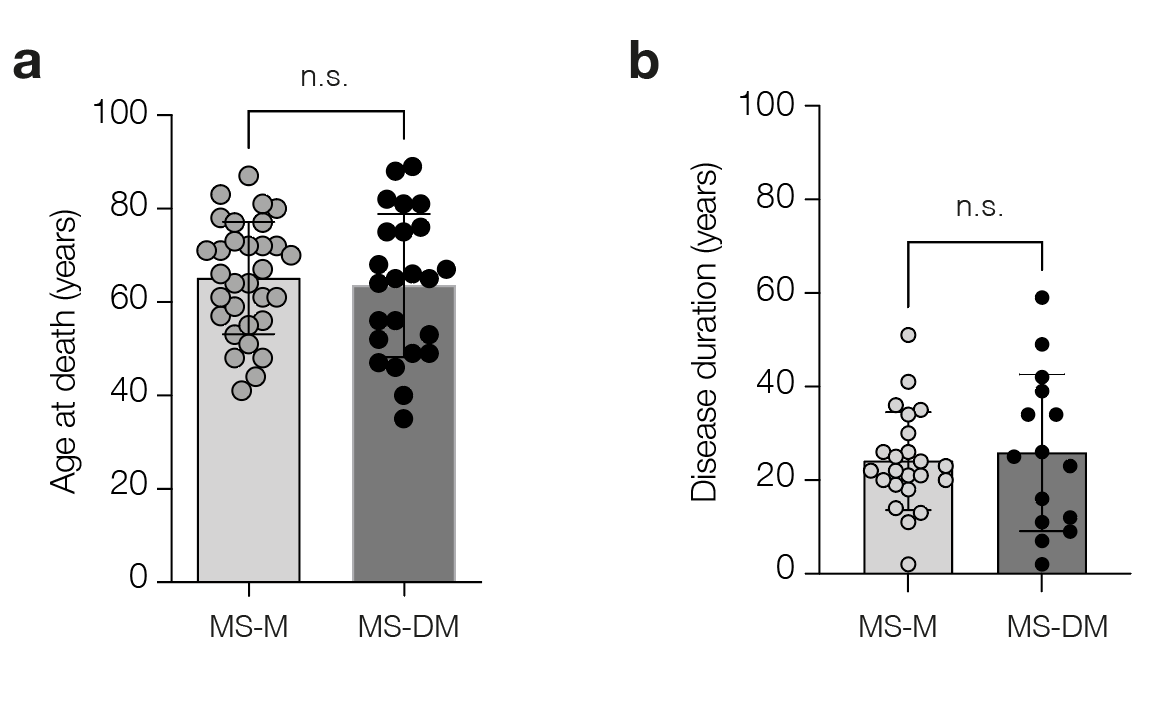
**

**Supplementary Figure 1. Clinical course parameters in MS cases without and with hippocampal demyelination.**

1. Age at death did not differ significantly between MS cases without and with hippocampal demyelination (Mann-Whitney U = 350.5, *P* = 0.72, *n* = 31 MS-M and *n* = 24 MD-DM).
2. Disease duration did not differ significantly between MS cases without and with hippocampal demyelination (Mann-Whitney U = 167, *P* = 0.87, *n* = 31 MS-M and *n* = 24 MD-DM).

**
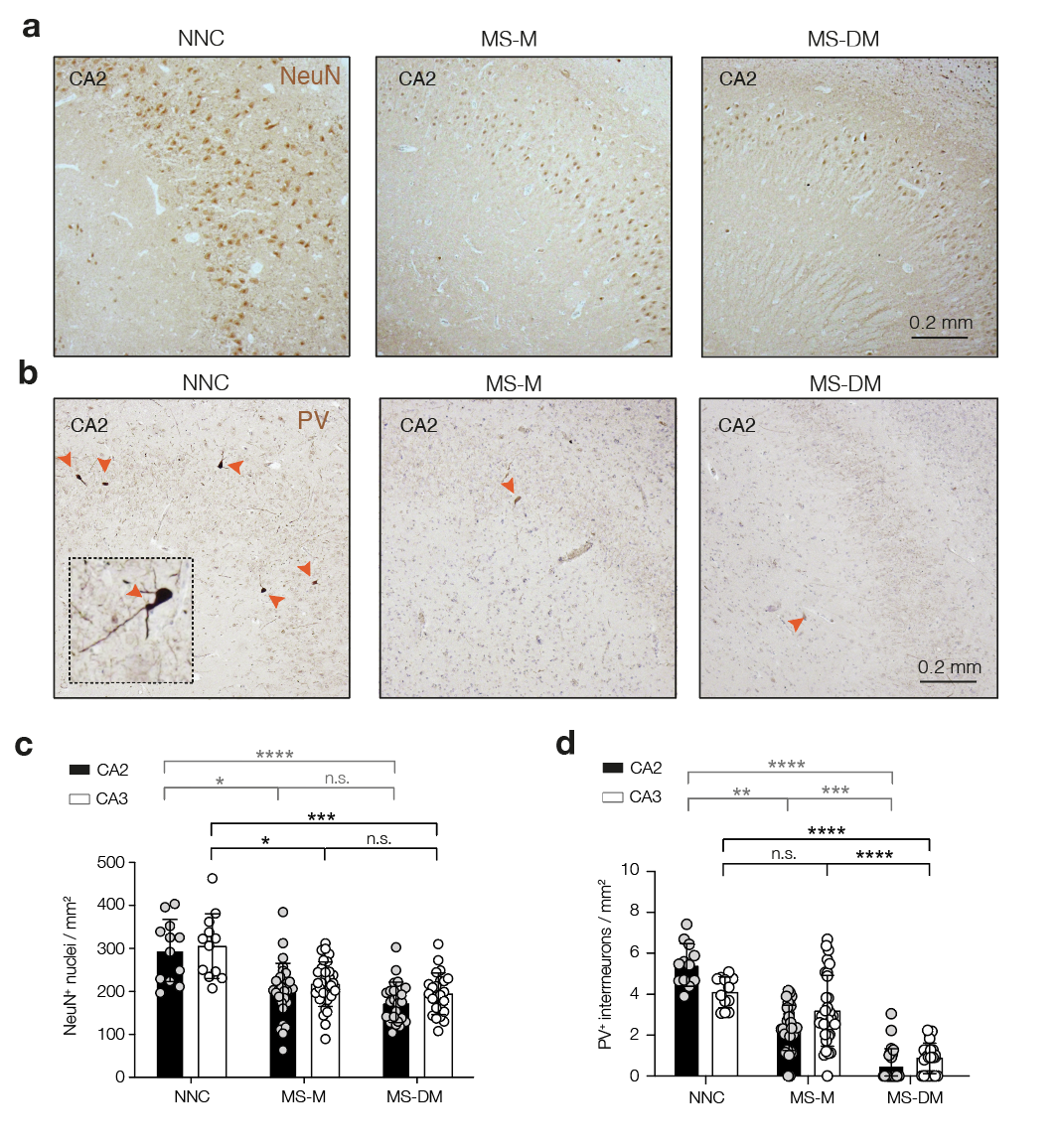
**

**Supplementary Figure 2. Loss of neurons and parvalbumin interneurons in the MS CA2.**

1. Immunohistochemistry for Neuronal Nuclei (NeuN) (in brown), identifying neurons, in the CA2 subfield of hippocampi from a non-neurological control case (NNC), an MS case with myelinated hippocampus (MS-M) and an MS case with demyelinated hippocampus (MS-DM). Scale bar, 0.2 mm
2. Immunohistochemistry for Parvalbumin (PV) (in brown), identifying parvalbumin interneurons (indicated by the red arrow an in the inset), in the CA2 subfield of hippocampi from a non-neurological control case (NNC), an MS case with myelinated hippocampus (MS-M) and an MS case with demyelinated hippocampus (MS-DM). Scale bar, 0.2 mm
3. Quantification of the density of NeuN^+^ nuclei in post-mortem hippocampal CA2 and CA3 subfields, showing a significant loss of neurons in both MS-M and MS-DM cases compared to NNC (Kruskal-Wallis test = 42.39, *P* < 0.0001, Dunn’s multiple comparison tests for CA2; NNC vs. MS-M **P* = 0.011, NNC vs MS-DM *****P* < 0.0001, MS-M vs. MS-DM *P* = 0.68. CA3; NNC vs MS-M **P* = 0.035, NNC vs MS-DM ****P* = 0.0006, MS-M vs MS-DM *P* > 0.99, *n* = 12 NNC, *n* = 31 MS-M and *n* = 24 MD-DM). Bar plots show mean ± SEM and circles represent individual hippocampi.
4. Quantification of the density of PV^+^ neurons in post-mortem hippocampal CA2 and CA3 subfields, reveals a significant loss of PV^+^ neurons in MS-M and a further aggravation of PV loss in the MS-DM cases compared to NNC (Kruskal-Wallis test = 86.26, *P* < 0.0001, followed by Dunn’s multiple comparison tests. CA2; NNC vs. MS-M ***P* = 0.0021, NNC vs MS-DM *****P* < 0.0001, MS-M vs. MS-DM ****P* = 0.0003. CA3; NNC vs MS-M *P* > 0.99, NNC vs MS-DM *****P* < 0.0001, MS-M vs MS-DM ****P* < 0.0001, *n* = 12 NNC, *n* = 31 MS-M and *n* = 24 MD-DM). Bar plots show mean ± SEM and circles represent individual hippocampi.

**
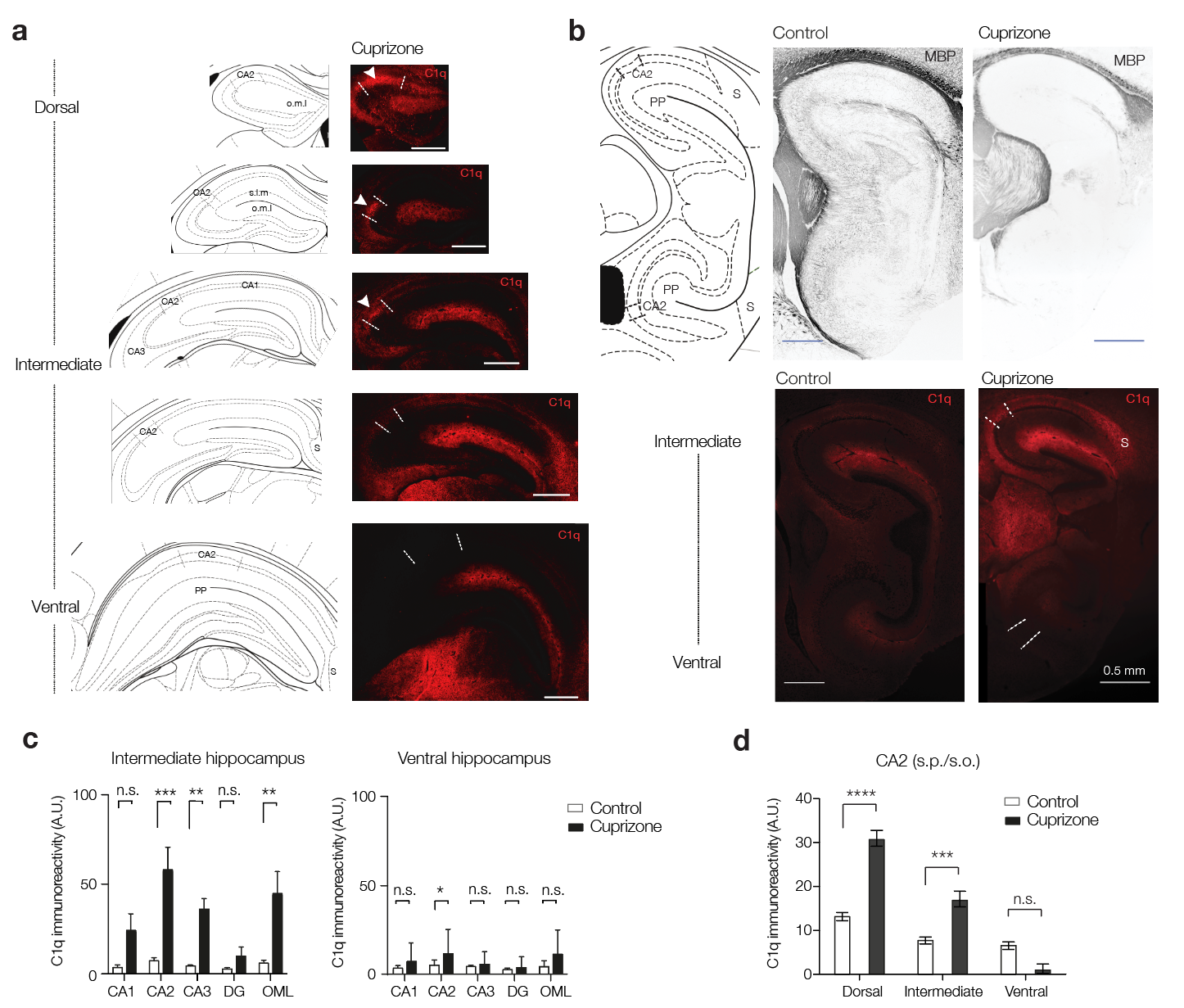
**

**Supplementary Figure 3. Dorsal to ventral gradient of cuprizone-induced C1q upregulation across the longitudinal axis of the hippocampus**

1. Left, coronal sections of the hippocampus adapted from the mouse brain atlas (Paxinos and Watson). Right, immunofluorescence images for C1q for the same locations shown on the left in cuprizone-treated mice (9 weeks, 0.2%). Note the high C1q intensity in the dorsal hippocampus (white arrows) but not in the ventral hippocampus (CA2 within dotted lines).
2. Sagittal view of the intermediate and ventral hippocampus. Note the difference between intermediate and ventral C1q intensity.
3. Population analysis of the hippocampal subfields for relative C1q intensity, as percentage area reveals a gradient in upregulation (two-way ANOVA with Sidak’s multiple comparison tests ****P* < 0.001, ***P* < 0.01, **P* < 0.05, n.s. = not significant, *n* = 6; 2 slices from 3 brains per group). Data shown as mean ± SEM. Closed bars, cuprizone and open bars control mice.
4. Comparative analysis of cuprizone-induced C1q upregulation across three anatomical levels of the CA2 subfield of the longitudinal axis of the hippocampus reveals a gradient in its increase (two-way ANOVA followed by Sidak’s post-hoc tests. Dorsal *****P* < 0.0001, intermediate ****P* = 0.0001, ventral *P* = 0.396, *n* = 6; 2 slices from 3 brains per group). C1q signals were averaged for the pyramidal and oriens layers. All data shows as mean ± SEM.

**
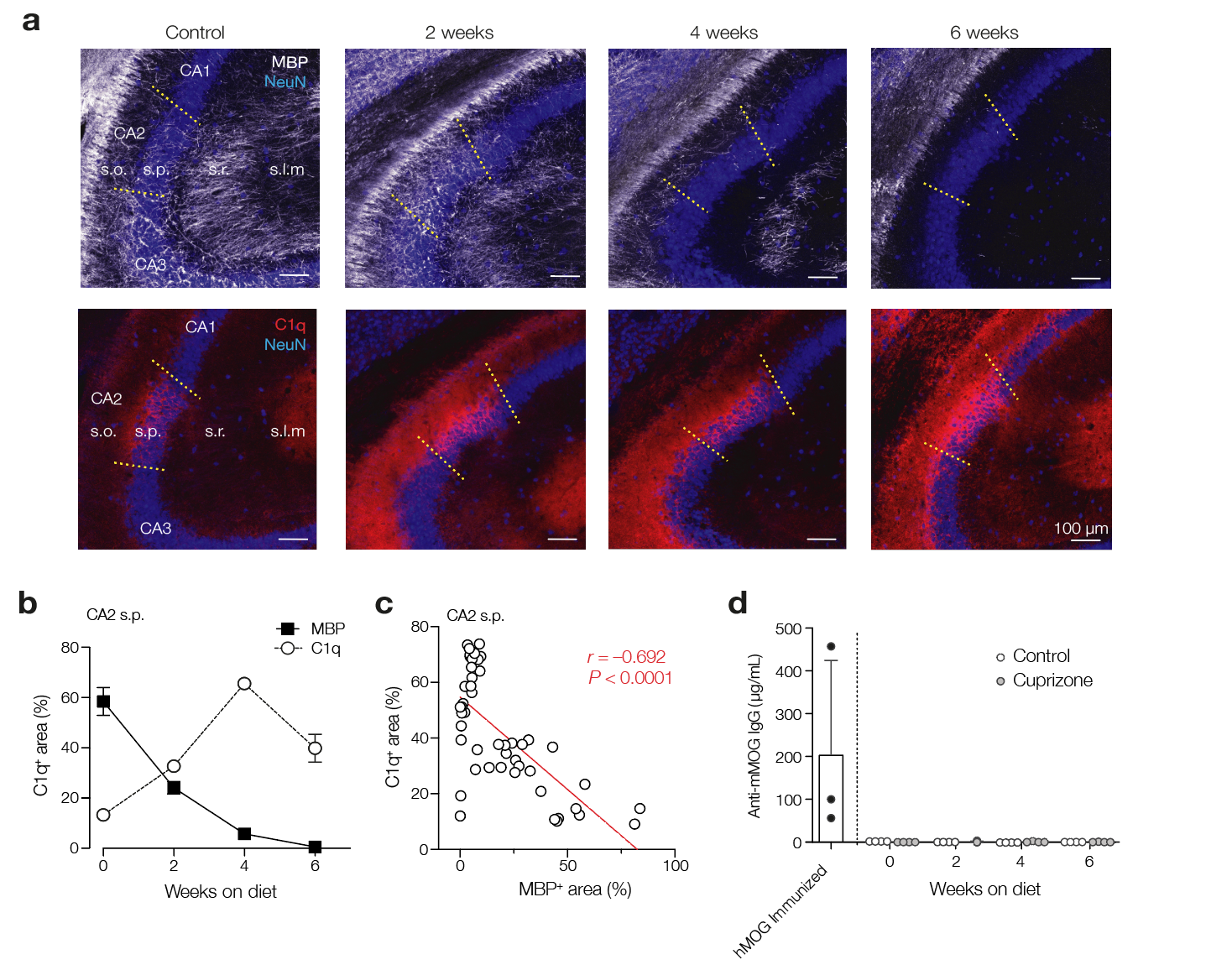
**

**Supplementary Figure 4. Temporal development of MBP and C1q in the CA2 subfield**

1. Example immunofluorescence images showing the dorsal CA2 hippocampal subfield for myelin basic protein (MBP, top, white) and C1q (bottom, red) overlaid with the neuronal marker NeuN (blue). The top and bottom row are the same brain sections from triple immunofluorescence staining separate for visual clarity. From left to right; control hippocampus and 2, 4 and 6-weeks treatment with the cuprizone diet.
2. Population data for the quantification of MBP and C1q intensities for the CA2 stratum pyramidale subfield in the dorsal hippocampus (48 images from 32 mice, 4 mice/group).
3. The cuprizone-induced MBP loss and C1q increase are negatively correlated across brain sections for the s.p. subfield (Pearson’s correlation coefficient *r* = –0.69, *n* = 48 sections from 4 mice per time/group). In contrast, neither in the stratum oriens (s.o.) nor in the stratum radiatium (s.r.) the myelin loss and C1q gain were significantly correlated (*P* = 0.160, slope = –0.350 and *P* = 0.102, slope = –0.0219, respectively, *n* = 48 sections from 4 mice/time/group).
4. ELISA was used to measure anti-mouse MOG IgG1 in the blood of control (*n* = 4) and cuprizone-fed (*n* = 4) mice throughout the period of cuprizone feeding up to 6 weeks. Sera from human myelin oligodendrocyte glycoprotein (MOG)-immunized C57BL/6 mice (*n* = 3) were collected during the chronic phase of experimental autoimmune encephalomyelitis (EAE) and used as an internal technical control

**
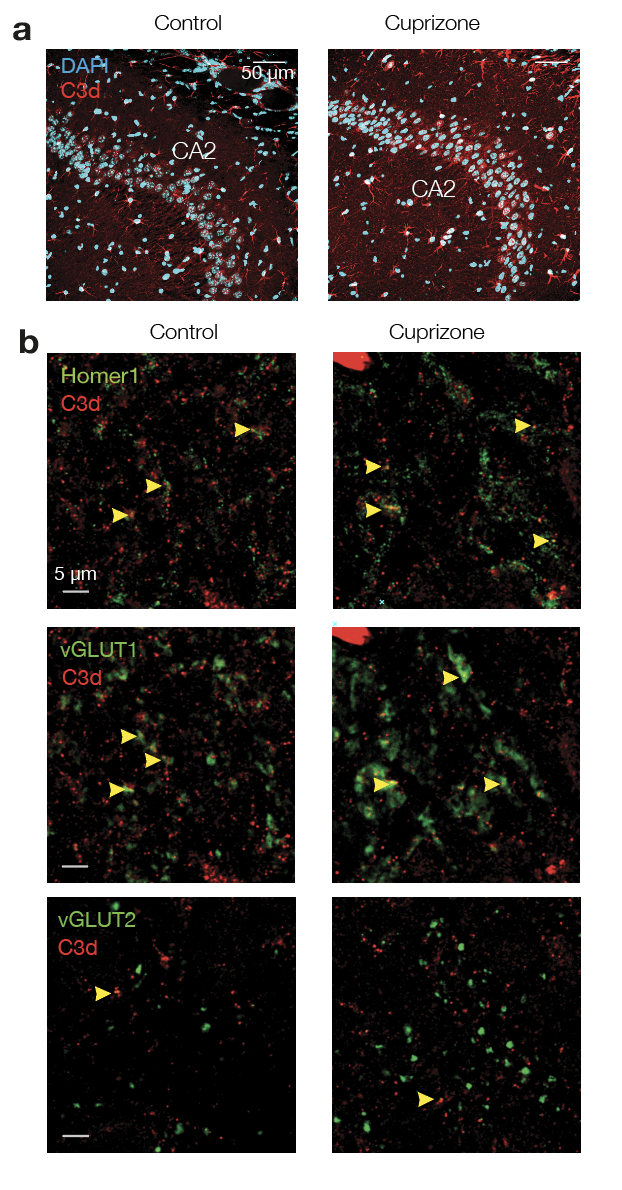
**

**Supplementary Figure 5. Demyelination-induced C3d upregulation show rare co-localization with synaptic elements.**

1. Double immunofluorescence staining for C3d (red) and DAPI (blue). Note that C3d immunoreactivity is detected in control hippocampus and increased in cuprizone hippocampus (see quantification of the C3d^+^ area in Figure 4 h, main manuscript).
2. Double immunofluorescence staining for C3d (red) and Homer1 or vGLUT1 or vGLUT2 (green) in CA2 area of control mice and mice after 9 wks, 0.2% cuprizone. Arrowheads indicate overlap in expression (yellow).

**
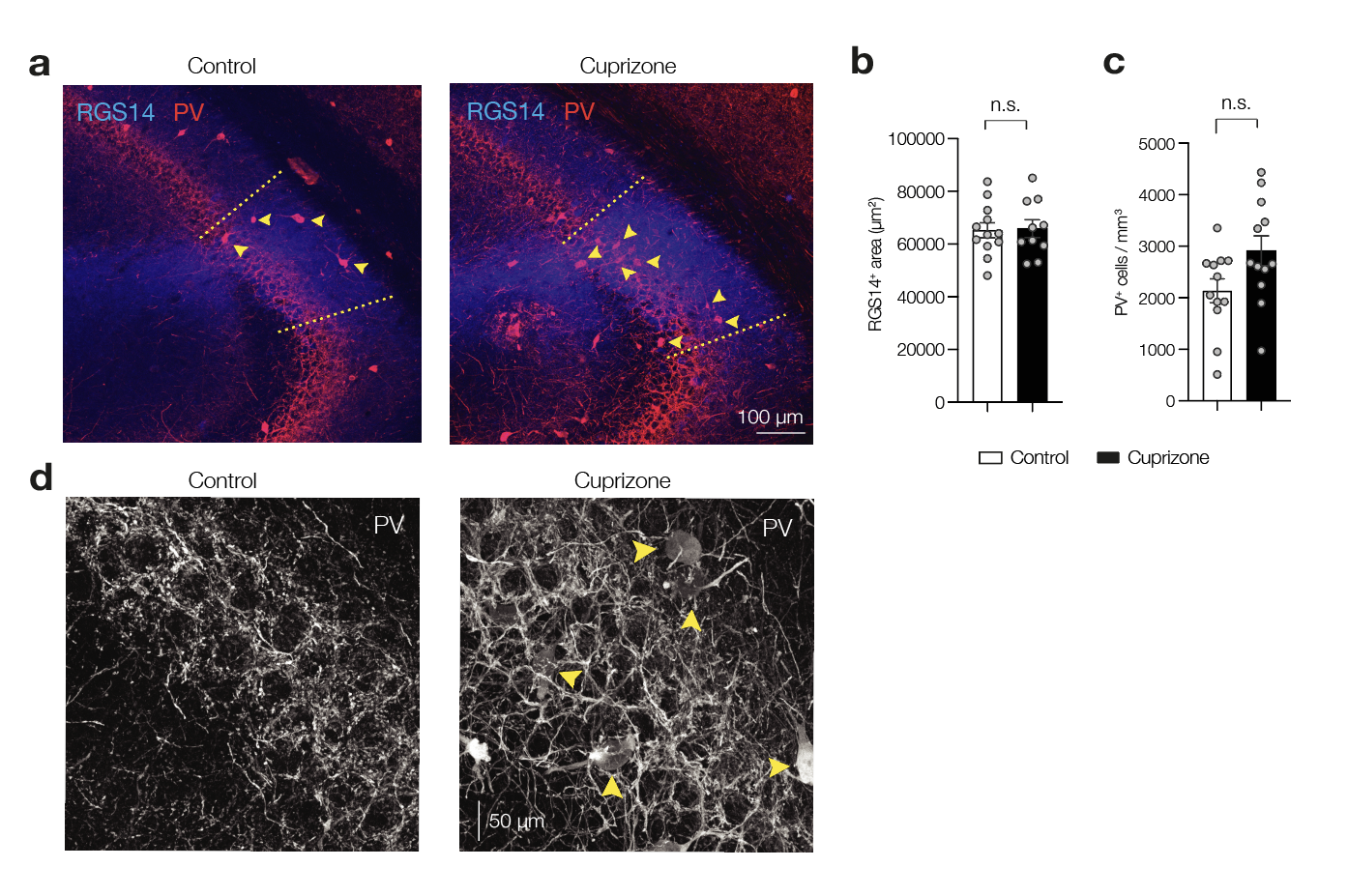
**

**Supplementary Figure 6. Cuprizone treatment does not affect the CA2 area or PV cell number.**

1. Double immunofluorescence for RGS14 (blue) and parvalbumin (anti-PV, red) in control hippocampus (left) and the hippocampus after 7-weeks 0.2% cuprizone treatment (right). The borders of the CA2 region are indicated with dashed yellow line. PV^+^ somata included in the analysis are indicated with yellow arrows.
2. Population analysis for the RGS14^+^ area as an indication for the CA2 field size shows no difference (two-tailed Mann-Whitney test U = 56, *P* = 0.377, *n* = 12 sections for 6 mice/group. For each hemisphere 10 consecutive sections of 40 µm in the left and right dorsal hippocampus were stained and averaged.
3. Population analysis for the PV^+^ neurons in CA2 reveals no change with cuprizone-induced demyelination (two-tailed Mann-Whitney test U = 43, *P* = 0.101, *n* = 12 sections for 6 mice/group). Mean ± SEM. Circles show individual hippocampus, left and right.
4. Higher magnification of the CA2 region and PV immunofluorescence (grey). Yellow markers indicate PV^+^ cell bodies.

**
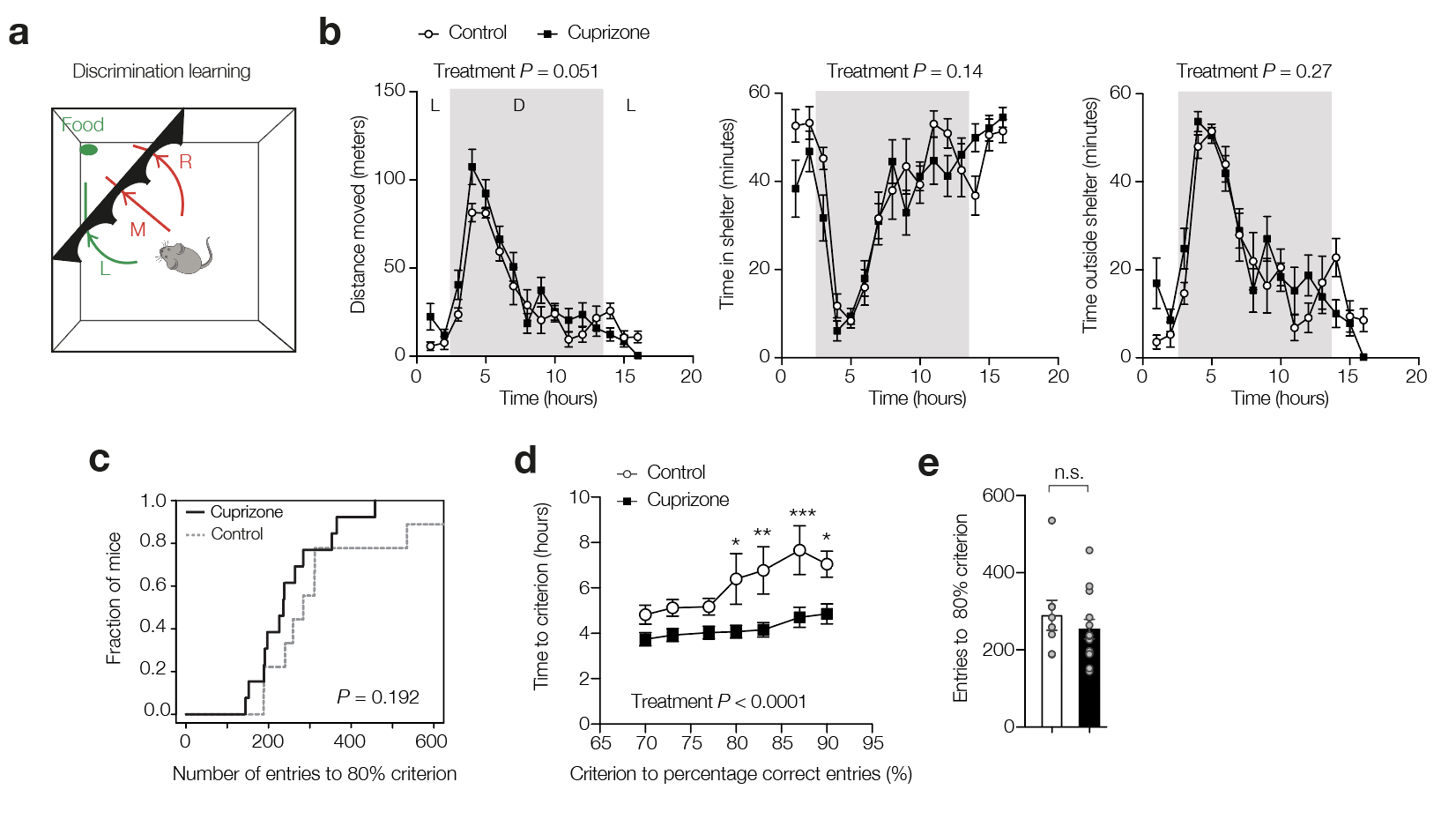
**

**Supplementary Figure 7. Cuprizone treatment does not affect general activity while improving spatial discrimination learning**

1. Schematic design of the automated PhenoTyper arrangement showing the cognition wall with three ports and an automated food pellet dispenser. Mice needed to engage in a continuously running task to be rewarded with a pellet food, automatically dispensed when the mouse ran 5 times through the left port (green line, discrimination stimulus).
2. One-hour binned plots of the parameters (from left to right) distance moved, time inside shelter and time outside the shelter box over 16 hours continuous recording of mice individually housed inside the PhenoTyper cage. Cuprizone treatment did not change activity patterns (distance moved; two-way ANOVA *F*_1, 20_ = 4.33, Treatment *P* = 0.051, Time × treatment *F*_15, 300_ = 1.71, *P* = 0.048). Time within the shelter; mixed-effect model *F*_1, 319_ = 2.14, *P* > 0.145 and time outside shelter; mixed-effect model *F*_1, 319_ = 1.093, *P* = 0.296, respectively, *n* = 9 control and *n* = 13 cuprizone mice). Data and connecting lines show mean ± SEM. Note that both groups follow similar patterns across light (L) and dark (D) phases of the circadian cycle.
3. Cumulative distribution plot of the fraction of mice versus total number of the total number of entries until 80% learning criterion. Learning criteria was assessed by a moving average over the last 30 entries in which a specific percentage (80%) of entries through the left (L) entry port (providing the food reward) was achieved. (Chi-square test = 1.85, *P* = 0.192, *n* = 9 control and *n* = 13 cuprizone mice).
4. Cuprizone-treated mice more rapidly learned to discriminate the cognition wall task. Time until a specific cut-off learning criterium was reached plotted against the learning criterion between 70 and 90% correct entries. Ordinary two-way ANOVA Treatment *F*_1, 132_ = 46.29, ****P* < 0.0001, Percentage *F*_6, 132_ = 3.85, ***P* = 0.0014, Interaction *F*_6, 132_ = 1.088, *P* = 0.37. Note the cuprizone treated mice reach the learning cut-off before control mice. Sidak’s multiple comparison tests for < 77% *P* > 0.6, for 80% **P* = 0.0157, 83% ***P* = 0.0043, 87% ****P* = 0.0008, 90% **P* < 0.0382 (for all, *n* = 8 control, *n* = 13 cuprizone mice).
5. The total number of entries through all three ports (L, M and R) until a 80% criterion was observed was not different (two-tailed unpaired *t*-test with Welch’s correction (*t, df*) = 0.775, 12.83; *P* = 0.451).

**Supplementary movies**

**Supplementary movie S1**

Example movie of a control mouse in the third trial within a five-trial social test. A familiar mouse is placed in the cage for the duration of 1 minute.

**Supplementary movie S2**

Example movie of a cuprizone-treated mouse in the third trial. The familiar mouse is placed in the cage for the duration of 1 minute. Note the long duration of anogenital sniffing and frequent approaching.

| **SUPPLEMENTARY TABLES**  **Supplementary Table 1. Clinical data of MS donors and controls** | | | | | | | |
| --- | --- | --- | --- | --- | --- | --- | --- |
| **Case** | **Sex**  M/F | **Age**  (years) | **PMD**  (hours) | **DD**  (years) | **MS type** | **Cognitive decline** | **COD** |
| MS |  |  |  |  |  |  |  |
| *Myelinated* |  |  |  |  |  |  |  |
| 1993-307 | F | 72 | 9.50 | 41 | PP | Unknown | Baroreflex insufficiency |
| 1995-276 | M | 56 | 5.75 | 13 | SP | Unknown | Respiratory insufficiency |
| 1996-264 | F | 67 | 4.90 | 2 | PP/SP | Unknown | Euthanasia |
| 1996-352 | F | 53 | 7.25 | 18 | SP | Unknown | Pneumonia |
| 1998-087 | F | 55 | 7.90 | 11 | PP/SP | Unknown | Aspiration pneumonia |
| 1998-185 | F | 70 | 8.90 | 19 | PP | Unknown | Cardiogenic shock & pneumonia |
| 1999-025 | F | 64 | 7.75 | 35 | SP | Unknown | Dehydration & pneumonia |
| 2000-124 | M | 64 | 7.50 | 34 | PP | Unknown | End stage MS |
| 2002-025 | M | 77 | 4.25 | 26 | SP | Unknown | Cerebral vascular accident |
| 2002-053 | F | 48 | 5.85 | 21 | PP/SP | Unknown | Congestive cardiac failure |
| 2003-106 | F | 72 | 8.85 | 20 | SP | Unknown | General deterioration |
| 2004-055 | M | 71 | 7.00 | 26 | PP/SP | Unknown | Pneumonia by aspiration |
| 2007-001’ | F | 57 | 23.05 | 21 | PP/SP | + | Sepsis |
| 2007-002’ | F | 78 | 11.10 | 30 | SP | - | Dehydration |
| 2007-071’ | M | 41 | 7.20 | 14 | PP | + | Urosepsis and pneumonia |
| 2009-036’ | M | 72 | 7.55 | 23 | SP | - | Pneumonia |
| 2009-103’ | F | 59 | 4.45 | 24 | SP | - | Euthanasia |
| 2010-024’ | M | 44 | 10.15 | 22 | SP | + | General deterioration |
| 2011-120’ | M | 73 | 8.45 | 51 | SP | + | Urosepsis |
| 2012-032’ | M | 80 | 9.45 | 36 | SP | + | Cachexia and pneumonia |
| 2013-047’ | F | 48 | 11.50 | 22 | SP | + | Respiratory failure |
| 2013-081* | M | 66 | 10.55 | 25 | PP | Unknown | Euthanasia |
| 2013-032’ | F | 87 | 9.30 | 20 | PP/SP | - | Dehydration and Renal insufficiency |
| 2014-052* | F | 61 | 4.15 | Unknown | PP/SP | Unknown | Euthanasia |
| 2014-057* | M | 61 | 5.15 | Unknown | PP/SP | Unknown | Euthanasia |
| 2015-020* | F | 71 | 3.52 | Unknown | PP/SP | Unknown | Euthanasia |
| 2015-022* | M | 83 | 3.00 | Unknown | PP/SP | Unknown | Pneumonia |
| 2015-064* | M | 51 | 5.30 | Unknown | PP/SP | Unknown | Pneumonia |
| 2015-070* | F | 77 | 4.00 | Unknown | PP/SP | Unknown | Pneumonia |
| 2016-017* | M | 61 | 5.09 | Unknown | PP/SP | Unknown | Euthanasia |
| 2017-083* | F | 81 | 2.30 | Unknown | PP/SP | Unknown | Anorexia |
|  |  |  |  |  |  |  |  |
| *Demyelinated* |  |  |  |  |  |  |  |
| 1992-187 | F | 35 | 5.75 | 11 | SP | Unknown | General decline |
| 1996-116 | F | 81 | 4.25 | 49 | SP | Unknown | Cachexia |
| 1996-076 | M | 46 | 3.75 | 23 | SP | Unknown | Pneumonia |
| 1997-123 | F | 89 | 5.90 | 12 | PP/SP | Unknown | Respiratory tract infection |
| 1998-059 | F | 76 | 4.60 | 2 | PP/SP | Unknown | Uremia; dehydration |
| 1999-086 | M | 81 | 8.85 | 59 | PP | Unknown | General deterioration |
| 2001-001 | F | 68 | 7.85 | 16 | PP/SP | Unknown | Pneumonia |
| 2001-137 | F | 56 | 6.90 | 9 | PP | Unknown | Urosepsis |
| 2003-105 | M | 66 | 7.50 | 26 | PP | Unknown | Unknown |
| 2004-017 | M | 49 | 8.00 | 25 | SP | Unknown | Pneumonia by MS |
| 2004-035 | M | 47 | 7.25 | 7 | SP | Unknown | Urosepsis with organ failure |
| 2008-053’ | F | 64 | 10.10 | 39 | SP | - | Urinary tract infection |
| 2008-096’ | F | 88 | 7.55 | 34 | PP | - | Exhaustion by chronic colitis ulcerosis |
| 2009-007’ | F | 67 | 9.15 | 42 | SP | - | Euthanasia |
| 2011-080’ | F | 56 | 8.25 | 34 | PP | + | Pneumonia |
| 2015-006* | F | 53 | 4.00 | Unknown | PP/SP | Unknown | Euthanasia |
| 2015-108* | F | 82 | 3.40 | Unknown | PP/SP | Unknown | Euthanasia |
| 2016-050* | F | 52 | 3.40 | Unknown | PP/SP | Unknown | Euthanasia |
| 2016-052* | F | 75 | 5.15 | Unknown | PP/SP | Unknown | Respiratory failure |
| 2016-074* |  | 65 | 4.15 | Unknown | PP/SP | Unknown | Stroke |
| 2017-001* | F | 40 | 2.30 | Unknown | PP/SP | Unknown | Euthanasia |
| 2017-068* | F | 49 | 3.55 | Unknown | PP/SP | Unknown | Pneumonia |
| 2017-139* | M | 75 | 4.00 | Unknown | PP/SP | Unknown | Euthanasia |
| 2017-161* | F | 65 | 3.50 | Unknown | PP/SP | Unknown | Fever |
|  |  |  |  |  |  |  |  |
| Controls |  |  |  |  |  |  |  |
| *Non-neurological* |  |  |  |  |  |  |  |
| 1990-009 | M | 67 | Unknown | - | - | - | Pulmonary embolisms |
| 1992-042 | M | 61 | 13.50 | - | - | - | Carcinoma of the esophagus |
| 1993-127 | M | 64 | Unknown | - | - | - | Pancreatic cancer |
| 1997-115 | M | 81 | 7.90 | - | - | - | Renal insufficiency & heart failure |
| 1998-003 | F | 80 | 7.00 | - | - | - | Pulmonary embolisms |
| 2005-068 | F | 50 | 4.15 | - | - | - | Metastasized cell bronchocarcinoma |
| 2008-027 | F | 50 | 6.85 | - | - | - | Metastasized breast carcinoma |
| 2009-095 | F | 61 | 8.80 | - | - | - | Euthanasia |
| 2012-002 | M | 55 | 15.00 | - | - | - | Intestinal ischemia |
| 2014-043 | F | 60 | 8.10 | - | - | - | Breast cancer |
| 2015-027 | F | 76 | 4.45 | - | - | - | Adenocarcinoma |
| 2018-121 | F | 68 | 3.30 | - | - | - | Pancreatic cancer |
| PMD, Post-mortem delay; DD, Disease duration; MS, Multiple Sclerosis; COD, Cause of death; PP, Primary progressive; SP, Secondary progressive; F, Female; M, Male; AD, Alzheimer’s Disease. The asterisk * indicates cases used for pathological/MRI correlation studies. The apostrophe’ indicates cases used for pathological/cognitive correlation studies. | | | | | | | |

| **Supplementary Table 2. Primary antibodies, dilution, source for immunohistochemistry** | | | | |  | |
| --- | --- | --- | --- | --- | --- | --- |
| Antigen | Clone | Dilution | Source | Identifier | |  |
| ***Mouse*** |  |  |  |  | |  |
| Complement complex 1 (C1q) | Monoclonal 4.8 | 1:000 | Abcam | Cat# ab182451  RRID: AB_2732849 | |  |
| NeuN | A60 | 1:1000 | Millipore | Cat# ABN90P  RRID: AB_2341095 | |  |
| DAPI |  | 1.5 µg/ml | Vector | Cat# 7F0515 | |  |
| Myelin Basic Protein (MBP) | 12 | 1:250 | Millipore | Cat# MAB386  RRID: AB_94975 | |  |
| vGAT1 | Polyclonal | 1:500 | Millipore | Cat# AB5062P  RRID: AB_2301998 | |  |
| vGLUT1 | Polyclonal | 1:1000 | Synaptic Systems GmbH | Cat# 135304  RRID: AB_887878 | |  |
| vGLUT2 | 8G9.2 | 1:200 | Millipore | Cat# MAB5504  RRID: AB_2187552 | |  |
| Homer1 | Polyclonal | 1:200  1:500 | Synaptic Systems GmbH | Cat# 160006  RRID: AB_2631222 | |  |
| RGS14 | N133/21 | 1:500 | Neuromab | Cat# 75-170  RRID: AB_2179931 | |  |
| Purkinje cell protein 4 (PCP4) | Polyclonal | 1:250 | Sigma | Cat# HPA005792  RRID: AB_1855086 | |  |
| Streptavidin, Alexa Fluor 488^TM^ Conjugate |  | 1:400 | Invitrogen | Cat# S32354  RRID: AB_2315383 | |  |
| C3d Complement | Polyclonal | 1:500 | Agilent | Cat# A006302  RRID: AB_578478 | |  |
| Iba-1 | EPR16589 | 1:500 | Abcam | Cat# ab178847  RRID: AB_2832244 | |  |
| GFAP | Polyclonal | 1:100 | Bio-connect | Cat# SC-6170  RRID: AB_641021 | |  |
| ***Human*** |  |  |  |  | |  |
| Proteolipid protein (PLP) | plpc1 | 0.3 μg/ml^a^ | Serotec | Cat# MCA839G  RRID: AB_2237198 | |  |
| Human Leukocyte Antigen (HLA) | CR3/43 | 8.6 µg/ml^a^ | Abcam | Cat# Ab55152  RRID: AB_944199 | |  |
| Parvalbumin (PV) | Polyclonal | 1:2000 | Swant | Cat# PV27  RRID: AB_2631173 | |  |
| Neuronal nuclei (NeuN) | A60 | 1:1000 | Millipore | Cat# ABN90P  RRID: AB_2341095 | |  |
| Vesicular glutamate transporter 1 (vGLUT1) | Polyclonal | 1:400^b^ | Synaptic  Systems | Cat# 135 304  RRID: AB_887878 | |  |
| Vesicular GABA transporter 1 (vGAT) | Polyclonal | 1:100^b^ | Synaptic  Systems | Cat# 131 006  RRID: AB_2619820 | |  |
| Post Synaptic Domain (PSD) 95 | D27E11 | 1:150^b^ | Cell  Signalling | Cat# 3450  RRID: AB_2292883 | |  |
| Gephyrin | mAb7a | 1:100^b^ | Synaptic  Systems | Cat# 147 021  RRID: AB_2232546 | |  |
| C1q | Monoclonal [34E2] | 1:100^b^ | Abcam | Cat # ab235454 | |  |
| Antigen retrieval of paraffin sections was performed by heat in ^a^ 0.05 M Tris buffered saline pH 7.6 or ^b^ 10 mM Tris/1 mM EDTA buffer pH 9. | | | | | | |
